# Commensal Leucothoidae (Crustacea, Amphipoda) of the Ryukyu Archipelago, Japan. Part II: sponge-dwellers

**DOI:** 10.3897/zookeys.166.2313

**Published:** 2012-01-20

**Authors:** Kristine N. White, James Davis Reimer

**Affiliations:** 1 Rising Star Program, Trans-disciplinary Organization for Subtropical Island Studies (TRO-SIS), University of the Ryukyus, 1 Senbaru, Nishihara, Okinawa, Japan 903-0213

**Keywords:** Leucothoidae, Ryukyus, Okinawa, Japan, new species, commensal, *Leucothoe akaoni*, *Leucothoe bise*, *Leucothoe daisukei*, *Leucothoe hashi*, *Leucothoe lecroyae*, *Leucothoe nagatekubi*, *Leucothoe nurunuru*, *Leucothoe ouraensis*, *Leucothoe togatta*, *Leucothoe toribe*, *Leucothoe zanpa*

## Abstract

Commensal leucothoid amphipods have been collected from the canals of their sponge hosts throughout the Ryukyu Archipelago, Japan. Eleven new species are described in the genus *Leucothoe* with valuable location data and host records. An identification key to sponge-dwelling Leucothoidae of the Ryukyu Archipelago is provided.

## Introduction

The Leucothoidae are a marine family of gammaridean amphipods that can be found inhabiting sessile invertebrate hosts worldwide. The family currently contains 146 species in five genera and can be divided into two clades (White 2011, White and Reimer 2012a). Leucothoids are typically found as endocommensal associates of sponges, ascidians, and bivalve mollusks, where they utilize the feeding current produced by their hosts to feed (White 2011, White and Reimer 2012a).

There are currently 14 Leucothoidae species reported from Japan, with only seven of these from the Ryukyu Archipelago (White and Reimer 2012a). A map of the study area is available in part 1 (White and Reimer 2012a).

## Methods

Specimens were collected via snorkeling and SCUBA at 47 locations throughout the Ryukyu Archipelago: Ishigaki–jima Island (4 locations), Iriomote–jima Island (4), Okinawa–jima Island (21), Yoron–to Island (2), Okinoerabu–jima Island (2), Tokunoshima Island (4), Amami–oshima Island (6), and Yakushima Island (4) (see map in White and Reimer 2012a). Detailed station data are available in Supplementary Table 1 in White and Reimer (2012a).

Entire sponges were isolated in zip-lock plastic bags for subsequent dissection in the laboratory or amphipods were captured individually in situ using a modified squirt bottle (following Thomas and Klebba 2006, 2007). When possible, pieces of sponge were preserved in 99% EtOH. Sponges were tentatively identified by Nicole de Voogd (Naturalis, Leiden) via photos of sponges and sponge spicules and by referencing Allen and Steene (2002). Coral rubble samples were also taken, elutriated, and sieved on location using both saltwater and formalin washes. Samples were sorted immediately. Amphipods were preserved in 2% seawater buffered formalin for morphological analysis and 99% ethanol for molecular studies.

Specimens used for morphological analyses were transferred to glycerin, dissected, mounted on slides, and illustrated using a Nikon®Y-IDT drawing tube attached to a Nikon® Eclipse 50I compound microscope. Pencil drawings were scanned and digitally inked in Adobe® Illustrator using a Wacom® Tablet, following the methods of Coleman (2003).

Descriptions are of males unless noted with sexually dimorphic characters described in a separate section. Terminology used in descriptions follows White and Thomas (2009) with ‘proximal margin’ of the carpus and dactylus referring to the margins closing on the propodus. Setae nomenclature follows Oshel and Steele (1988) where possible without having SEM images for the specimens described here. All setae are simple, unless noted.

Type material and sponge pieces are deposited in the University of the Ryukyus Museum (Fujukan), with the prefix RUMF for museum numbers. Additional material has been deposited in the National Museum of Nature and Science in Tokyo, with the prefix NSMT for museum numbers.

Scale bars in figures represent 0.1 mm unless noted.

Figures legend: **Hd** head; **Mx** maxilla; **Md** mandible; **Xpd** maxilliped; **LL** lower lip; **UL** upper lip; **G** gnathopod; **P** pereopod; **T** telson; **U** uropod; **L** left; **R** right; **l** lateral; **m** medial; **p** paratype; **+** enlarged.

## Taxonomy

### Leucothoe

Leach, 1814

http://species-id.net/wiki/Leucothoe\according_to_White_et_al_2012

#### Generic diagnosis.

Eyes, if present, generally well developed with 10 or more ommatidia. Mandibles lacking molars, palp three articulate; right lacinia mobilis smaller than left. Maxilliped inner plates fused, palp 4–articulate; outer plates not reaching apex of palp article 1. Coxa 1–4 relatively equal in widths. Pereopods 5–7 bases generally expanded. Minimal to no sexual dimorphism.

#### Leucothoe akaoni sp. n.

urn:lsid:zoobank.org:act:F254D541-1FB7-432B-A62E-665D65E787B6

http://species-id.net/wiki/Leucothoe_akaoni

Figures 1 2

##### Type material.

Holotype female, 9.6 mm RUMF-ZC-1732, Zanpa Cape, Okinawa–jima Island, Okinawa, reef wall (26°26'27"N ,127°43'03"E), in canals of large white ball sponge, Tetillidae of Sollas 1886, 30 m, Daisuke Ueno, col., 26 February 2011 (KNWOkinawa34C). Paratype male, 8.3 mm, RUMF-ZC-1733, Zanpa Cape, Okinawa–jima Island, Okinawa, reef wall (26°26'27"N, 127°43'03"E), in canals of large white ball sponge, 30–33 m, K.N. White and N.S. White, col., 3 April 2011 (KNWOkinawa40D).

##### Type locality.

Zanpa Cape, Okinawa–jima Island, Okinawa, Japan (26°26'27"N, 127°43'03"E).

##### Additional material examined.

1 specimen, RUMF-ZC-1734, KNW17Aug10; 1 specimen, NSMT-Cr 21871, KNWOkinawa10A; 3 specimens, NSMT-Cr 21872, KNWOkinawa15G; 2 specimens, RUMF-ZC-1735, KNWOkinawa16I; 2 specimens, RUMF-ZC-1736, KNWOkinawa29D; 0.5 specimen, NSMT-Cr 21873, KNWOkinawa34C; 15 specimens, NSMT-Cr 21874, KNWYaku2F; 1 specimen, RUMF-ZC-1737, KNWOkinawa40D.

##### Diagnosis (female).

Ventral cephalic keel anteroventral margin with anteriorly projecting cusp. Right mandible lacinia mobilis with 2 rows of dentition. Maxilliped inner plates with short serrate robust setae. Gnathopod 1 coxa anterior margin serrate; basis anterior margin with 12 medium-length setae, posterior margin with 14 short setae. Gnathopod 2 basis anterior margin with 33 medium-length setae, posterior margin with 7 setae, distal margin with 4 curved setae; ischium with several short posterior and distal setae and posterodistal serrations; carpus distally truncate.

##### Description (female).

Head. Anterior margin rounded, anterodistal margin evenly rounded; ventral cephalic keel anterior margin excavate, anteroventral margin with anteriorly projecting cusp, ventral margin straight; eyes present with more than 10 ommatidia, oval. Antenna 1 0.3 × body length, flagellum 10–articulate, peduncle article 1 width less than 2 × article 2, accessory flagellum absent. Antenna 2 0.3 × body length, subequal in length with antenna 1, flagellum 8–articulate. Mandibular palp ratio of articles 1–3 1.0: 2.6: 1.7, article 2 with 8–9 long distal setae, article 3 with 2 distal setae, incisors strongly dentate; left mandible with 16 raker spines, lacinia mobilis large, strongly toothed; right mandible with 14 raker spines, lacinia mobilis small, with 2 rows of dentition. Upper lip asymmetrically lobate, anterior margin setose. Lower lip inner lobes fused, bare; outer lobes with small gape, anterior margins setose. Maxilla 1 palp 2–articulate with 4 distal setae; outer plate with 9 distal robust setae and 4 distal slender setae. Maxilla 2 inner plate with 8 slender distal setae, 6 robust marginal setae, and facial setae; outer plate with 2 robust and 6 slender distal setae, 9 slender marginal setae, and facial setae. Maxilliped inner plates distal margin with a v-shaped indentation, with short serrate robust setae; outer plate inner margin smooth, reaching 0.3 × palp article 1, with simple marginal setae, facial setae present; palp article 4 subequal in length with article 3, distally acute.

Pereon. Coxae 1–4 relative widths 1.0: 1.3: 1.0: 1.4. Gnathopod 1 coxa with tiny marginal setae, anterior margin serrate, anterodistal margin produced, rounded, distal margin straight, posterior margin excavate, facial setae absent; basis slightly inflated, anterior margin with 12 short setae, posterior margin with 14 short setae; ischium with posterior setae; carpus linear, distal length 12.9 × width, proximal margin dentate, distal margin with 3 setae; propodus straight, palm dentate with 8 distal setae; dactylus smooth, reaching 0.5 × propodus length. Gnathopod 2 coxa broader than long, subequal in size with coxa 3, smooth, with tiny marginal setae; anterior margin straight, anterodistally rounded, distal margin straight, posterior margin straight, facial setae absent; basis slightly posteriorly expanded, anterior margin with 33 medium setae, posterior margin with 7 setae, distal margin with 4 curved setae; ischium with several short posterior and distal setae and posterodistal serrations; carpus 0.3 × propodus length, curved, distally truncate, anterior margin dentate; propodus with 1 mediofacial setal row displaced to midline, reaching 0.8 × propodus length, with 1 row of submarginal setae, posterior margin smooth, palm convex dentate; dactylus curved, proximal margin smooth, bare, anterior margin distally subacute, reaching 0.6 × propodus length. Pereopod 3 coxa length 1.3 × width, anterodistal corner overriding distal face of coxa 2 and extending below it, smooth, bare, anterior margin straight, distal margin oblique, posterior margin tapered, facial setae absent. Pereopod 4 coxa smooth, bare, anterior margin straight, distal margin evenly rounded, posterior margin tapered, facial setae absent. Pereopods 5–7 coxae facial setae absent; bases width length ratios 1: 1.4, 1: 1.4, 1: 1.3, posterior margins smooth, setose.

Pleon. Epimera 1 and 3 bare, epimeron 2 with ventral setae; epimeron 3 posteroventral corner subquadrate. Uropods 1–3 relative lengths 1.0: 0.8: 1.1. Uropod 1 peduncle and outer ramus 0.9 × inner ramus length; inner ramus with 3 robust setae; outer ramus with 7 robust setae. Uropod 2 peduncle length 0.7 × inner ramus length, outer ramus length 0.6 × inner ramus length; inner ramus with 4 robust setae; outer ramus with 7 robust setae. Uropod 3 peduncle 1.3 × inner ramus length, outer ramus length subequal in length with inner ramus; inner ramus with 4 robust setae; outer ramus with 5 robust setae. Telson 2.7 × longer than wide, apex strongly tridentate.

**Male (sexually dimorphic characters).**

Gnathopod 1 basis anterior margin with 7 setae, posterior margin with 5 setae. Gnathopod 2 basis anterior margin with 9 long setae, posterior margin bare; ischium with posterodistal setae, smooth.

**Figure 1.**
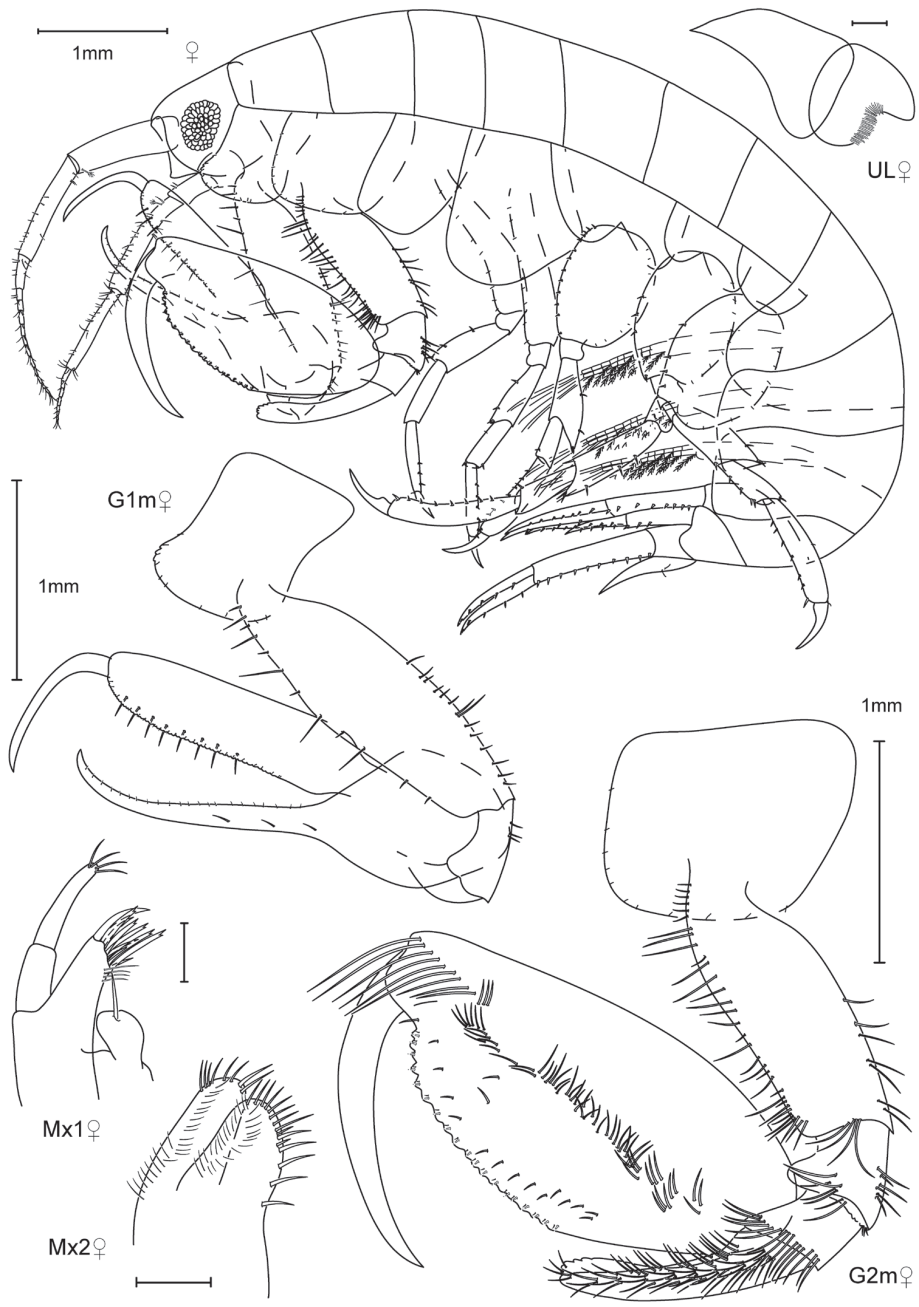
*Leucothoe akaoni* sp. n., holotype female, 9.6 mm, RUMF-ZC-1732.

**Figure 2.**
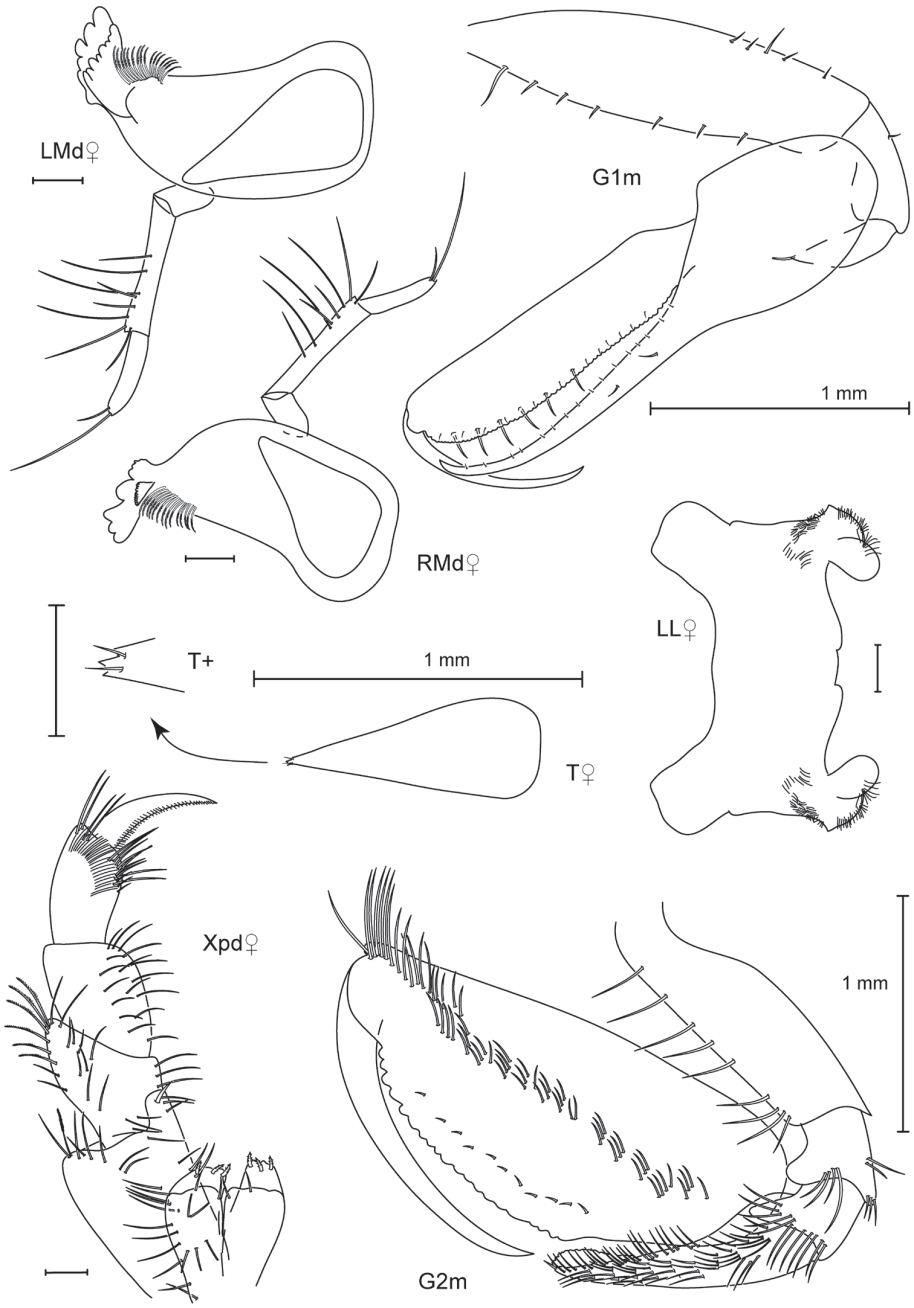
*Leucothoe akaoni* sp. n., holotype female, 9.6 mm, RUMF-ZC-1732; paratype male, 8.3 mm, RUMF-ZC-1733.

##### Etymology.

After the Japanese words ‘aka’, meaning ‘red’, and ‘oni’, meaning ‘barbarian’ and referring to the red color and large size (pronounced ah-ka-oh-nee).

##### Ecology.

In canals of sponges, spiculose green ball sponge, ?*Axinyssa* of Lendenfeld 1897 (Figure 24E); spiculose beige sponge, Niphatidae of Van Soest 1980 (probably *Niphates* of Duchassaing and Michelotti 1864), RUMF-ZP-6, KNWOkinawa10A (Figure 25I); large white ball sponge, Tetillidae, RUMF-ZP-11, KNWOkinawa40E (Figure 24I); and among coral rubble.

##### Relationships.

*Leucothoe akaoni* sp. n. is similar to *Leucothoe denticulata* Costa, 1851 and *Leucothoe wuriti* Thomas & Klebba, 2007 in having a rounded head, ventral cephalic keel with projection, gnathopod 1 dactylus reaching greater than 0.2 × propodus length, and a truncate gnathopod 2 carpus. *Leucothoe akaoni* sp. n. differs from these species in lacking facial setae on coxa 1, having a setose posterior margin on the gnathopod 1 basis, gnathopod 2 basis anterior margin with more than 9 setae, slightly less broadly expanded bases on pereopods 5–7.

##### Remarks.

*Leucothoe akaoni* sp. n. is deep red in color, darkest on the head, fading to deep orange posteriorly (Figure 23A). This species has been collected only on Yakushima Island, Kagoshima and from the northwestern coast of Okinawa–jima Island, Okinawa.

##### Distribution.

East China Sea: Okinawa–jima Island (Okinawa), Yakushima (Kagoshima), Japan.

#### Leucothoe bise sp. n.

urn:lsid:zoobank.org:act:316E130F-9811-4DAE-9666-4625146E5339

http://species-id.net/wiki/Leucothoe_bise

Figures 3 4

##### Type material.

Holotype male, 2.1 mm RUMF-ZC-1738, Bise, Okinawa–jima Island, Okinawa, reef wall (26°42'46"N, 127°52'44"E), among coral rubble, 7–15 m, K.N. White and N.S. White, col., 9 February 2011 (KNWOkinawa30D). Paratype female, 3.6 mm RUMF-ZC-1739, same station data as holotype. Paratype male, 3.3 mm RUMF-ZC-1846, Yona, Kunigami, Okinawa–jima Island, Okinawa, reef wall (26°45'56"N, 128°46'39"E), among coral rubble, 9 m, K.N. White and N.S. White, col., 23 October 2010 (KNWOkinawa15E).

##### Type locality.

Bise, Okinawa–jima Island, Okinawa, Japan (26°42'46"N, 127°52'44"E).

##### Additional material examined.

1 specimen, NSMT-Cr 21875, KNWOkinawa23F; 1 specimen, NSMT-Cr 21876, KNWOkinawa27E; 1 specimen, RUMF-ZC-1740, KNWOkinawa29B; 1 specimen, RUMF-ZC-1741, KNWOkinawa29M; 6 specimens, RUMF-ZC-1742, KNWOkinawa30D; 1 specimen, NSMT-Cr 21877, KNWOkinawa31G; 5 specimens, NSMT-Cr 21878, KNWOkinawa39I; 6 specimens, NSMT-Cr 21879, KNWOkinawa43C; 1 specimen, NSMT-Cr 21880, KNWOkinawa50C; 1 specimen, RUMF-ZC-1743, KNWOkinawa55A; 5 specimens, RUMF-ZC-1795, KNWYaku3G; 1 specimen, RUMF-ZC-1847, KNWOkinawa15E; 1 specimen, RUMF-ZC-1848, KNWOkinawa15E; 4 specimens, NSMT-Cr 21991, KNWOkinawa15E; 1 specimen, RUMF-ZC-1849, KNWOkinawa21C; 2 specimens, NSMT-Cr 21992, KNWOkinawa13E.

##### Diagnosis (male).

Head anterodistal margin quadrate with cusp. Antenna 1 with many setulate-serrate setae. Maxilla 1 palp 1–articulate, margins constricted. Gnathopod 1 basis proximally widened; propodus palm serrate, saw-like. Gnathopod 2 carpus distally rounded; propodus with 2 mediofacial setal rows, primary mediofacial setal row very short. Pereopods 5–7 bases very narrowly expanded. Female gnathopod 1 basis posterior margin with 7 setae; gnathopod 2 carpus distally truncate.

##### Description (male).

Head. Anterior margin truncate, anterodistal margin quadrate with cusp; ventral cephalic keel anterior margin transverse, anteroventral margin quadrate, ventral margin straight; eyes with more than 10 ommatidia, round. Antenna 1 0.3 × body length, with setulate-serrate setae, flagellum 4–articulate, peduncle article 1 width less than 2 × article 2, accessory flagellum absent. Antenna 2 0.3 × body length, subequal in length with antenna 1, flagellum 3–articulate. Mandibular palp ratio of articles 1–3 1.0: 3.0: 1.8, article 2 with 5 medium distal setae, article 3 with 2 distal setae, incisors strongly dentate; left mandible lacinia mobilis with 12 raker spines, large, strongly toothed; right mandible with 10 raker spines, lacinia mobilis small, strongly dentate. Upper lip asymmetrically lobate, anterior margin setose. Lower lip inner lobes fused, bare; outer lobes with moderate gape, anterior margins setose. Maxilla 1 palp 1–articulate, margins constricted and with 4 distal setae; outer plate with 5 distal robust setae and 4 distal setae. Maxilla 2 inner plate with 5 robust distal setae; outer plate with 3 robust distal setae and 11 slender distal marginal setae. Maxilliped inner plates distal margin with a v-shaped indentation, with short robust setae; outer plate inner margin smooth, reaching 0.2 × palp article 1, with simple marginal setae, facial setae absent; palp 4–articulate, article 4 subequal in length with article 3, distally acute.

Pereon. Coxae 1–4 relative widths 1.0: 1.0: 0.9: 1.1. Gnathopod 1 coxa smooth, with tiny marginal setae, anterodistal margin produced, subtriangular, distal margin straight, posterior margin excavate, facial setae absent; basis proximally widened, anterior margin bare, posterior margin with 2 setae; ischium bare; carpus linear, distal length 18.8 × width, proximal margin smooth, distal margin bare; propodus straight, palm serrate, saw-like, with 3 robust and 5 short proximal setae; dactylus smooth, reaching 0.4 × propodus length. Gnathopod 2 coxa broader than long, subequal in size to coxa 3, smooth, with tiny marginal setae, anterior margin straight, anterodistally acute, distal and posterior margins straight, facial setae absent; basis linear, anterior margin with 3 setae, posterior margin bare; ischium bare; carpus 0.3 × propodus length, curved, distally rounded, anterior margin smooth; propodus with 2 mediofacial setal rows, primary mediofacial setal row above midline, reaching 0.4 × propodus length, secondary mediofacial setal row with 1 seta, with 1 row of submarginal setae, posterior margin smooth, palm convex, dentate; dactylus curved, proximal margin smooth, with 2 setae, anterior margin distally subacute, reaching 0.6 × propodus length. Pereopod 3 coxa length 0.9 × width, anterodistal corner overriding distal face of coxa 2 and extending below it, smooth, with tiny marginal setae, anterior margin evenly rounded, distal margin slightly convex, posterior margin straight, facial setae absent. Pereopod 4 coxa smooth, with tiny marginal setae, anterior margin tapered, distal margin straight, posterior margin tapered, facial setae absent. Pereopods 5–7 coxae facial setae absent; bases width length ratios 1: 1.9, 1: 1.7, 1: 1.8, posterior margins smooth, setose.

Pleon. Epimera 1 and 3 bare, epimeron 2 with ventral setae; epimeron 3 posteroventral corner subquadrate, produced. Uropods 1–2 relative lengths 1.0: 0.9; inner and outer rami lined with short marginal setae. Uropod 1 peduncle 0.9 × inner ramus length, outer ramus subequal in length with inner ramus length; inner ramus with 2 robust setae, outer ramus with 1 robust seta. Uropod 2 peduncle 0.7 × inner ramus length, outer ramus 0.9 × inner ramus length; inner ramus with 1 robust seta, outer ramus with 2 robust setae. Uropod 3 missing. Telson 2.1 × longer than wide, with plumose facial setae, apex weakly tridentate.

**Female (sexually dimorphic characters).**

Gnathopod 1 basis anterior margin with 1 seta, posterior margin with 7 setae; ischium with 2 posterior setae; carpus distal margin with 3 setae. Gnathopod 2 basis anterior margin with 10 medium setae, posteromedial margin with 1 seta; carpus distally truncate, reaching 0.5 × propodus length; propodus secondary mediofacial setal row with 4 setae, palm with more prominent tubercles.

**Figure 3.**
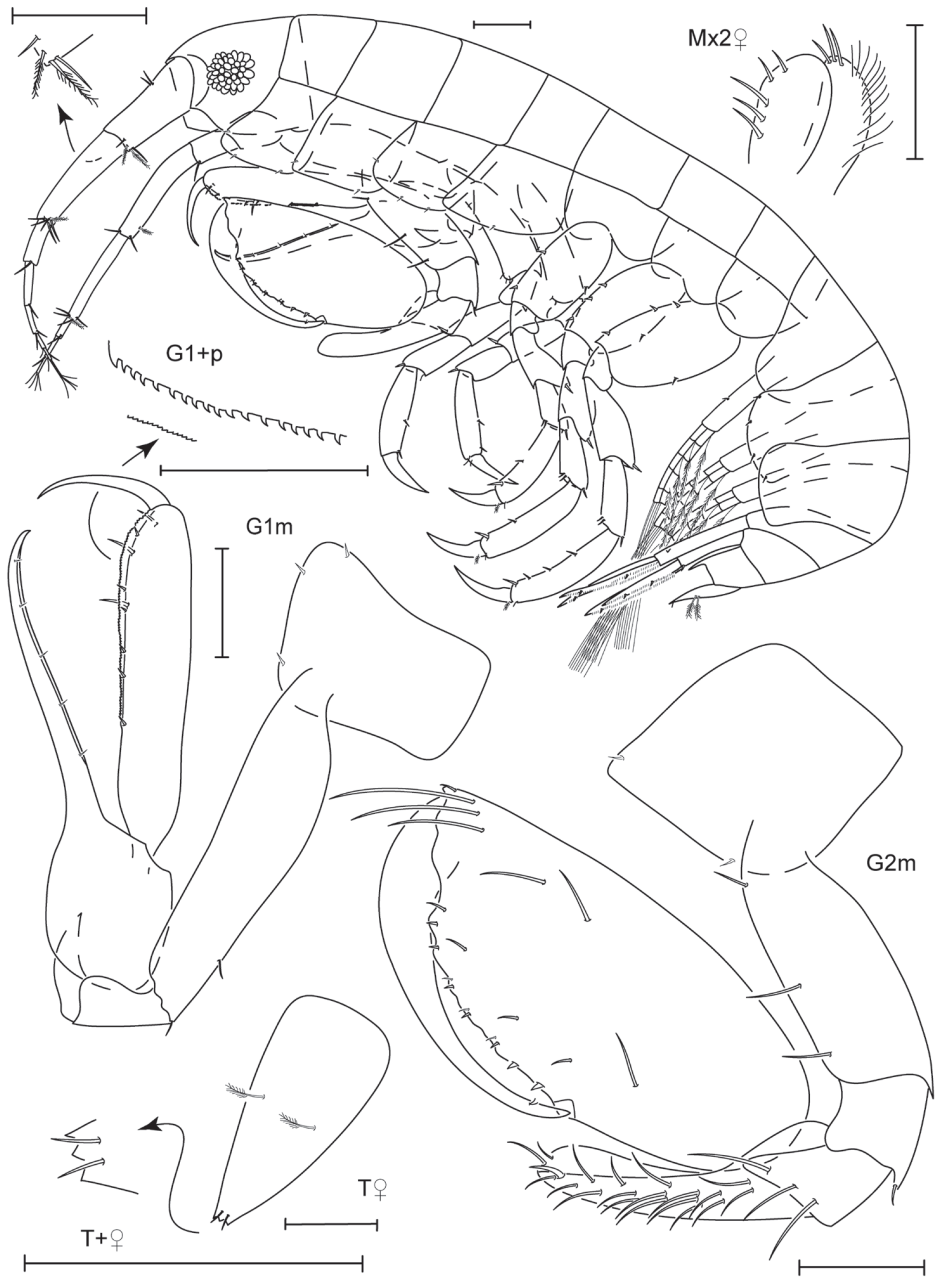
Leucothoe bise sp. n., holotype male, 2.1 mm, RUMF-ZC-1738; paratype female, 3.6 mm, RUMF-ZC-1739; paratype male, 3.3 mm RUMF-ZC-1846.

**Figure 4.**
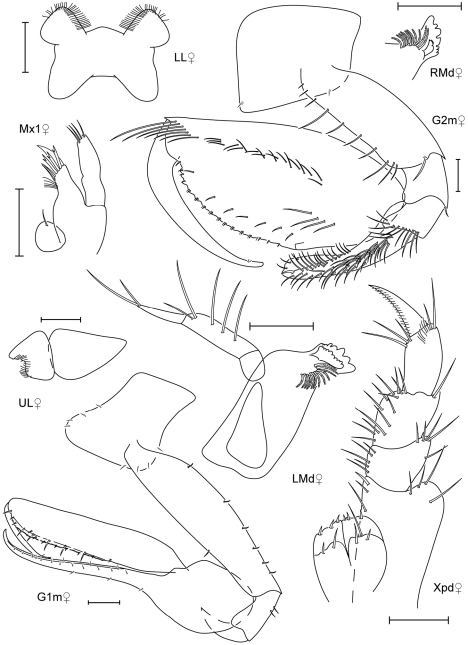
*Leucothoe bise* sp. n., paratype female, 3.6 mm, RUMF-ZC-1739.

##### Etymology.

After the Japanese place name, ‘Bise’, and referring to the type locality. (Pronounced bee-say.)

##### Ecology.

In canals of sponges, *Tedania* of Gray 1867 (Figure 25E); and among coral rubble.

##### Relationships.

*Leucothoe bise* sp. n. is similar to *Leucothoe hortapugai* Winfield et al., 2009 and *Leucothoe kensleyi* Thomas & Klebba, 2006 in having a quadrate anterior head margin, anterodistal head margin quadrate with cusp, narrow pereopod 5–7 bases, and gnathopod 1 dactylus reaching less than 0.2 × propodus. It differs from these species in having a quadrate keel without a projection, maxilla 1 palp 1–articulate, margins constricted, bare gnathopod 1 basis anterior margin, gnathopod 2 carpus distally rounded and reaching less than 0.4 × propodus length, and a produced posteroventral margin on epimeron 3.

##### Remarks.

Some sexually dimorphic characters may be due to the larger size of the female. Some specimens have larger serrations on the saw-like gnathopod 1 propodus palm, suggesting morphological variation in this character, perhaps depending on size. *Leucothoe bise* sp. n. is translucent orange in color (Figure 24B). This species has been collected on Iriomote–jima and throughout the western coast of Okinawa–jima Island, Okinawa.

##### Distribution.

East China Sea: Okinawa–jima Island, Iriomote–jima Island (both Okinawa), Japan.

#### Leucothoe daisukei sp. n.

urn:lsid:zoobank.org:act:93EDC549-9973-448C-A5E0-88ED1BD45443

http://species-id.net/wiki/Leucothoe_daisukei

Figures 5 6

##### Type material.

Holotype male, 4.4 mm RUMF-ZC-1744, Zanpa Cape, Okinawa–jima Island, Okinawa, reef wall (26°26'27"N, 127°43'03"E), in canals of large white ball sponge, Tetillidae, 30 m, Daisuke Ueno, col., 26 February 2011 (KNWOkinawa34F). Paratype female, 2.8 mm RUMF-ZC-1745, Zanpa Cape, Okinawa–jima Island, Okinawa, reef wall (26°26'27"N, 127°43'03"E), among coral rubble, 10–30 m, K.N. White and N.S. White, col., 26 February 2011 (KNWOkinawa34N).

##### Type locality.

Zanpa Cape, Okinawa–jima Island, Okinawa, Japan (26°26'27"N, 127°43'03"E).

##### Additional material examined.

2 specimens, KNWOkinawa34E; 2 specimens, KNWIshigaki2C; 1 specimen, RUMF-ZC-1746, KNWOkinawa40E; 1 specimen, NSMT-Cr 21881, KNWOkinawa34F.

##### Diagnosis (male).

Body stout. Antennae robust, short. Ventral cephalic keel anterior margin excavate. Mandibular palp article 2 robust. Maxilla 1 palp 1–articulate. Maxilliped inner plates with short serrate robust setae. Pereopods 5 and 7 coxae with facial setae. Gnathopod 2 stout.

##### Description (male).

Head. Anterior margin rounded, anterodistal margin subquadrate; ventral cephalic keel anterior margin excavate, anteroventral margin subquadrate, ventral margin oblique; eyes with more than 10 ommatidia, round. Antenna 1 0.3 × body length, flagellum 6–articulate, peduncle article 1 width less than 2 × article 2, accessory flagellum absent. Antenna 2 0.3 × body length, subequal in length with antenna 1, flagellum 6–articulate. Mandibular palp ratio of articles 1–3 1.0: 2.3: 1.8, article 2 robust, with 2–3 long distal setae, article 3 with 2 distal setae, incisors strongly dentate; left mandible with 11 raker spines, lacinia mobilis large, strongly toothed; right mandible with 11 raker spines, lacinia mobilis small, strongly dentate. Upper lip asymmetrically lobate, anterior margin setose. Lower lip inner lobes fused, setose; outer lobes with moderate gape, anterior margins setose. Maxilla 1 palp 1–articulate with 4 distal setae; outer plate with 4 distal robust setae and 10 distal setae. Maxilla 2 inner plate with 7 robust distal setae; outer plate with 3 robust distal setae and 16 slender distal marginal setae. Maxilliped inner plates distal margin with a v-shaped indentation, with short and long serrate robust setae; outer plate inner margin smooth, reaching 0.2 × palp article 1, with simple marginal setae, facial setae absent; palp article 2 with setulate-serrate marginal setae; article 4 subequal in length with article 3, distally acute.

Pereon. Coxae 1–4 relative widths 1.0: 1.2: 0.8: 1.3. Gnathopod 1 coxa smooth, with tiny marginal setae, anterodistal margin produced, rounded, distal margin straight, posterior margin excavate, facial setae absent; basis linear, anterior margin with 3 setae, posterior margin with 1 seta; ischium bare; carpus linear, distal length 12.8 × width, proximal margin dentate, distal margin with 2 short setae; propodus straight, palm dentate with 4 long and 8 short distal setae; dactylus smooth, reaching 0.5 × propodus length. Gnathopod 2 coxa broader than long, subequal in size with coxa 3, smooth, with tiny marginal setae, anterior margin rounded, anterodistally rounded, distal margin straight, posterior margin straight, facial setae absent; basis distally expanded, anterior margin with 7 long setae, posterior margin with 3 setae, posteromedial margin with 1 long seta; ischium with distal, posterior, and posterodistal setae; carpus 0.4 × propodus length, curved, distally tapered, anterior margin smooth; propodus with 1 mediofacial setal row displaced to midline, reaching 0.8 × propodus length, with 1 row of submarginal setae, posterior margin smooth, palm convex, dentate; dactylus curved, proximal margin smooth with 2 setae, anterior margin distally acute, reaching 0.8 × propodus length. Pereopod 3 coxa length 1.4 × width, anterodistal corner overriding distal face of coxa 2 and extending below it, smooth, with tiny marginal setae, anterior margin straight, distal margin slightly convex, posterior margin straight, facial setae absent. Pereopod 4 coxa smooth, bare, anterior margin straight, distal margin evenly rounded, posterior margin tapered, facial setae absent. Pereopods 5 and 7 coxae facial setae present, pereopod 6 coxa facial setae absent. Pereopods 5–7 bases width length ratios 1: 1.3, 1: 1.1, 1: 1.1, posterior margins smooth, setose.

Pleon. Epimera 1 and 3 bare, epimeron 2 with ventral setae; epimeron 3 posteroventral corner subquadrate. Uropods 1–3 relative lengths 1.0: 0.8: 1.1. Uropod 1 peduncle and inner ramus 0.8 × inner ramus length; inner ramus with 2 robust setae, outer ramus with 1 robust seta. Uropod 2 peduncle 0.6 × inner ramus length, outer ramus 0.9 × inner ramus length; inner and outer rami each with 1 robust seta. Uropod 3 peduncle 1.1 × inner ramus length, outer ramus length 0.9 × inner ramus length; inner and outer rami lined with short marginal setae, inner ramus with 1 robust seta, outer ramus with 2 robust setae. Telson 2.1 × longer than wide, apex weakly tridentate.

**Female (sexually dimorphic characters).**

Gnathopod 1 basis anterior margin with 1 seta, posterior margin with 2 setae; carpus distal margin with 1 short seta; propodus palm with 2 long and 7 short distal setae; dactylus with 2 proximal setae. Gnathopod 2 basis anterior margin with 4 long setae, posterior margin with 2 setae, posteromedial margin with 1 medium seta; carpus anterior margin dentate.

**Figure 5.**
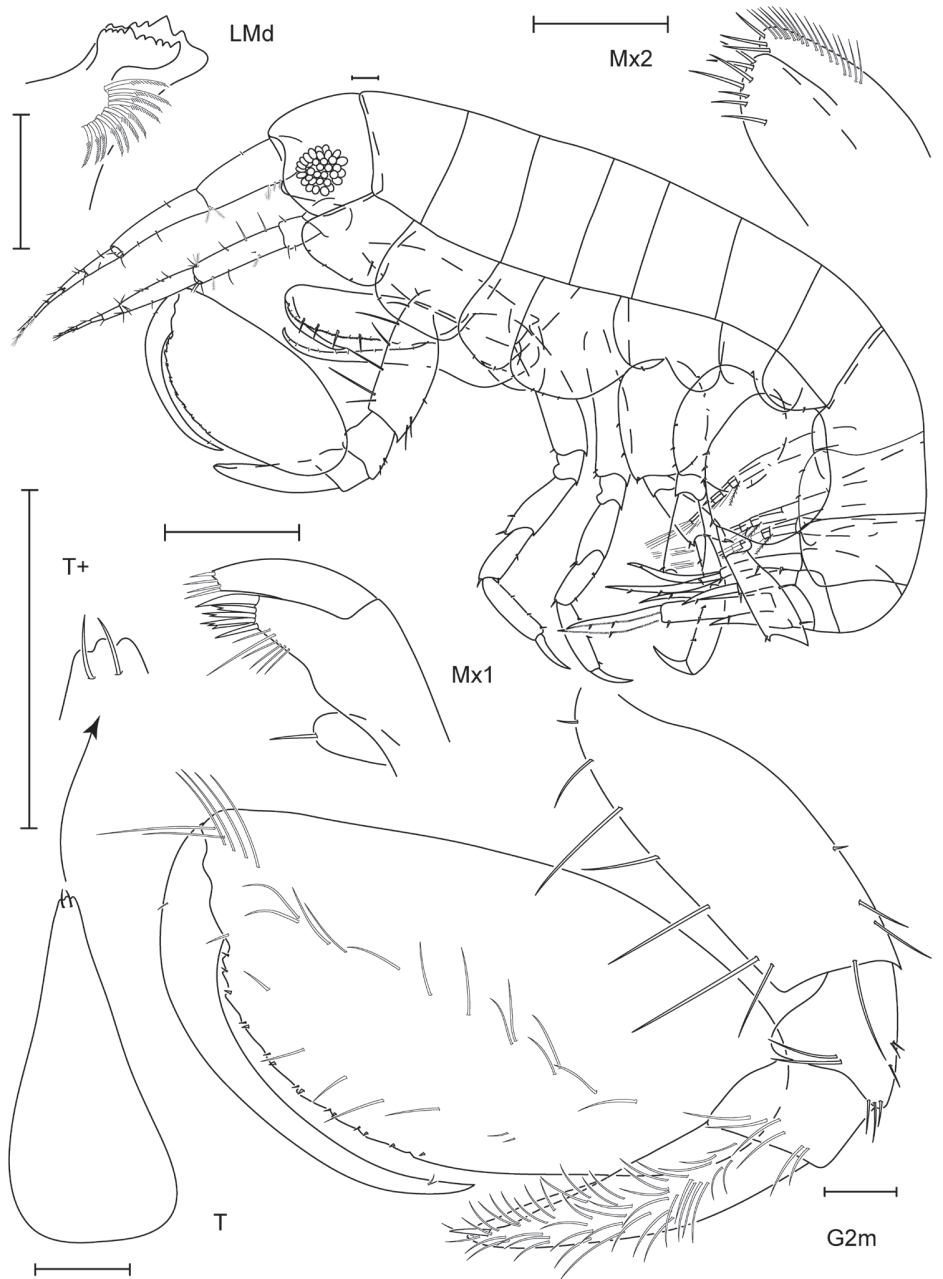
*Leucothoe daisukei* sp. n., holotype male, 4.4 mm, RUMF-ZC-1744.

**Figure 6.**
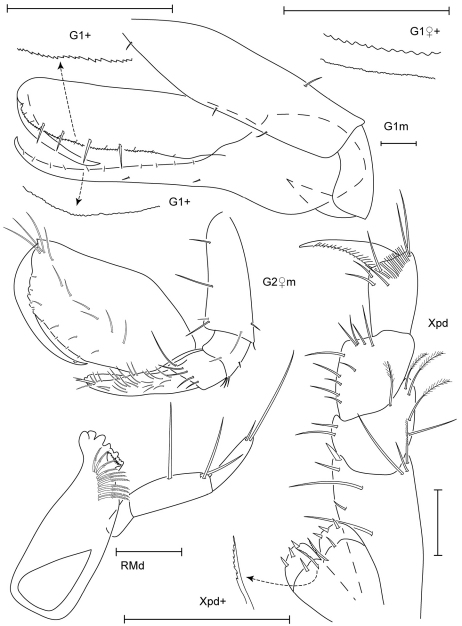
*Leucothoe daisukei* sp. n., holotype male, 4.4 mm, RUMF-ZC-1744; paratype female, 2.8 mm, RUMF-ZC-1745.

##### Etymology.

Named for Dr. Daisuke Ueno, who collected the type specimens of this species. Dr. Ueno has shared valuable specimens and collection locations on Okinawa–jima Island.

##### Ecology.

In canals of large white ball sponge, Tetillidae, RUMF-ZP-11, KNWOkinawa40E (Figure 24I); and among coral rubble.

##### Relationships.

*Leucothoe daisukei* sp. n. is similar to *Leucothoe madrasana* Sivaprakasam, 1969 in having a subquadrate anterodistal head margin and gnathopod 1 dactylus reaching greater than 0.2 × propodus length. *Leucothoe daisukei* sp. n. differs from this species in having a rounded anterior head margin, 1–articulate maxilla 1 palp, a smooth gnathopod 1 carpus proximal margin, and a tapered gnathopod 2 carpus distal margin.

##### Remarks.

*Leucothoe daisukei* sp. n. is peach in color, darkest along pereonite edges (Figure 23F). This species has been collected only on Ishigaki–jima Island and from 30 meters on the west coast of Okinawa–jima Island, Okinawa.

##### Distribution.

East China Sea: Okinawa–jima Island, Ishigaki–jima Island (both Okinawa), Japan.

#### Leucothoe hashi sp. n.

urn:lsid:zoobank.org:act:40C1B3A0-33C9-419C-A5D8-267DA462931C

http://species-id.net/wiki/Leucothoe_hashi

Figures 7 8

##### Type material.

Holotype male, 2.5 mm RUMF-ZC-1747, Mizugama, Okinawa–jima Island, Okinawa, reef wall (26°21'35"N, 127°44'22"E), in canals of yellow-beige sponge, *Callyspongia* of Duchassaing and Michelotti 1864, 8–10 m, K.N. White, col., 10 April 2011 (KNWOkinawa42A). Paratype female, 2.5 mm RUMF-ZC-1748, same station data as holotype. Paratype male, 1.9 mm RUMF-ZC-1749, Kaichu Doro, Okinawa–jima Island, Okinawa, seagrass bed (26°19'56"N, 127°55'23"E), in canals of green, hard branching sponge, ?*Clathria (Thalysias) reinwardti* Vosmaer, 1880, 1 m, K.N. White, col., 21 August 2010 (KNW_21Aug10).

##### Type locality.

Mizugama, Okinawa–jima Island, Okinawa, Japan (26°21'35"N, 127°44'22"E).

##### Additional material examined.

1 specimen, RUMF-ZC-1750, KNW21Aug10; 2 specimens, RUMF-ZC-1751, KNWOkinawa11B; 1 specimen, RUMF-ZC-1752, KNWOkinawa12H; 1 specimen, NSMT-Cr 21882, KNWOkinawa12H; 1 specimen, RUMF-ZC-1753, KNWOkinawa16D; 1 specimen, RUMF-ZC-1754, KNWOkinawa21B; 1 specimen, NSMT-Cr 21883, KNWOkinawa21E; 1 specimen, RUMF-ZC-1755, KNWOkinawa22B; 1 specimen, NSMT-Cr 21884, KNWOkinawa25C; 1 specimen, NSMT-Cr 21885, KNWOkinawa29C; 2 specimens, NSMT-Cr 21886, KNWOkinawa31B; 4 specimens, RUMF-ZC-1756, KNWOkinawa31F; 3 specimens, RUMF-ZC-1757, KNWOkinawa33E; 1 specimen, NSMT-Cr 21887, KNWOkinawa33E; 3 specimens, NSMT-Cr 21888, KNWOkinawa34G; 4 specimens, RUMF-ZC-1758, KNWOkinawa42A; 2 specimens, NSMT-Cr 21889, KNWOkinawa43A; 3 specimens, NSMT-Cr 21890, KNWOkinawa44A; 2 specimens, RUMF-ZC-1759, KNWIriomote2H; 1 specimen, NSMT-Cr 21891, KNWOkinawa51C; 1 specimen, RUMF-ZC-1760, KNWOkinawa53A; 2 specimens, NSMT-Cr 21892, KNWOkinawa53D; 1 specimen, RUMF-ZC-1761, KNWYaku5O; 2 specimens, RUMF-ZC-1762, KNWTokuno4C; 2 specimens, RUMF-ZC-1796, KNWYaku3J; 1 specimen, RUMF-ZC-1797, KNWYaku5I; 2 specimens, RUMF-ZC-1798, KNWOkinawa43D.

##### Diagnosis (male).

Antenna 1 accessory flagellum 1–articulate. Mandibular palp article 3 shorter than article 1. Maxilla 1 palp 1–articulate. Maxilliped outer plate tuberculate. Gnathopod 1 carpus and propodus very slender, chopstick-like; carpus proximal margin with denticles; propodus palm serrate with triangular teeth. Gnathopod 2 propodus with 2 mediofacial setal rows. Pereopods 5–7 bases narrowly expanded.

##### Description (male).

Head. Anterior margin rounded, anterodistal margin evenly rounded; ventral cephalic keel anterior margin slightly excavate, anteroventral margin subquadrate, ventral margin straight; eyes with more than 10 ommatidia, round. Antenna 1 0.3 × body length, flagellum 5–articulate, peduncle article 1 width less than 2 × article 2, accessory flagellum 1–articulate. Antenna 2 0.2 × body length, slightly shorter than antenna 1, flagellum 5–articulate. Mandibular palp ratio of articles 1–3 1.0: 2.0: 0.4, article 2 with 2 medium distal setae, article 3 with 1 distal seta, incisors weakly dentate; left mandible with 6 raker spines, lacinia mobilis large, strongly toothed; right mandible with 6 raker spines, lacinia mobilis small, strongly dentate. Upper lip asymmetrically lobate, anterior margin setose. Lower lip inner lobes fused, bare; outer lobes with moderate gape, anterior margins setose. Maxilla 1 palp 1–articulate with 4 distal setae; outer plate with 5 distal robust setae. Maxilla 2 inner plate with 5 robust distal setae and 2 robust facial setae; outer plate with 3 robust distal setae and 4 slender distal marginal setae. Maxilliped inner plates distal margin with a v-shaped indentation, with short robust setae; outer plate inner margin tuberculate, reaching 0.1 × palp article 1, with simple marginal setae, facial setae absent; palp article 4 subequal in length with article 3, distally acute.

Pereon. Coxae 1–4 relative widths 1.0: 1.2: 1.2: 1.3. Gnathopod 1 coxa smooth, with tiny marginal setae, anterodistal margin produced, rounded, distal margin straight, posterior margin slightly excavate, facial setae absent; basis distally expanded, anterior margin bare, posterior margin with 2 short setae; ischium bare; carpus linear, distal length 15.8 × width, proximal margin with denticles, distal margin bare; propodus straight, palm serrate with 7 distal triangular teeth and 7 distal setae; dactylus with small proximal notch, reaching 0.2 × propodus length. Gnathopod 2 coxa as long as broad, subequal in size with coxa 3, smooth, with tiny marginal setae, anterior margin expanded, anterodistally rounded, distal and posterior margins straight, facial setae absent; basis distally expanded, anterior margin with 5 medium setae, posterior margin with 1 seta; ischium with 1 posterodistal seta; carpus 0.3 × propodus length, curved, distally truncate, anterior margin dentate; propodus with 2 mediofacial setal rows, primary mediofacial setal row above midline, reaching 0.8 × propodus length, secondary mediofacial setal row with 2 setae, with 1 row of submarginal setae, posterior margin smooth, palm convex with 4 small denticles; dactylus curved, proximal margin smooth with 1 seta, anterior margin distally subacute, reaching 0.8 × propodus length. Pereopod 3 coxa length 1.0 × width, anterodistal corner overriding distal face of coxa 2 and extending below it, smooth, with tiny marginal setae, anterior margin straight, distal margin slightly convex, posterior margin straight, facial setae absent. Pereopod 4 coxa smooth, with tiny marginal setae, anterior margin straight, distal margin evenly rounded, posterior margin tapered, facial setae absent. Pereopods 5–7 coxae facial setae absent; bases width length ratios 1: 1.5, 1: 1.5, 1: 1.7, posterior margins smooth, setose.

Pleon. Epimera 1–2 with ventral setae, epimeron 3 bare; epimeron 3 posteroventral corner rounded. Uropods 1–3 relative lengths 1.0: 0.8: 1.2; inner and outer rami lined with short marginal setae. Uropod 1 peduncle and outer ramus 0.8 × inner ramus length; inner ramus with 6 robust setae and outer ramus with 5 robust setae. Uropod 2 peduncle 0.7 × inner ramus length, outer ramus 0.6 × inner ramus length; inner ramus with 3 robust setae and outer ramus with 4 robust setae. Uropod 3 peduncle 1.2 × inner ramus length, outer ramus 0.9 × inner ramus length; inner ramus with 4 robust setae and outer ramus with 7 robust setae. Telson 2.0 × longer than wide, apex weakly tridentate, almost bidentate.

**Female (sexually dimorphic characters).**

Gnathopod 1 basis posterior margin with 5 short setae. Gnathopod 2 basis posterior margin with 4 medium setae; ischium with 2 posterior and1 posterodistal seta; carpus distally tapered; propodus secondary mediofacial setal row with 4 setae.

**Figure 7.**
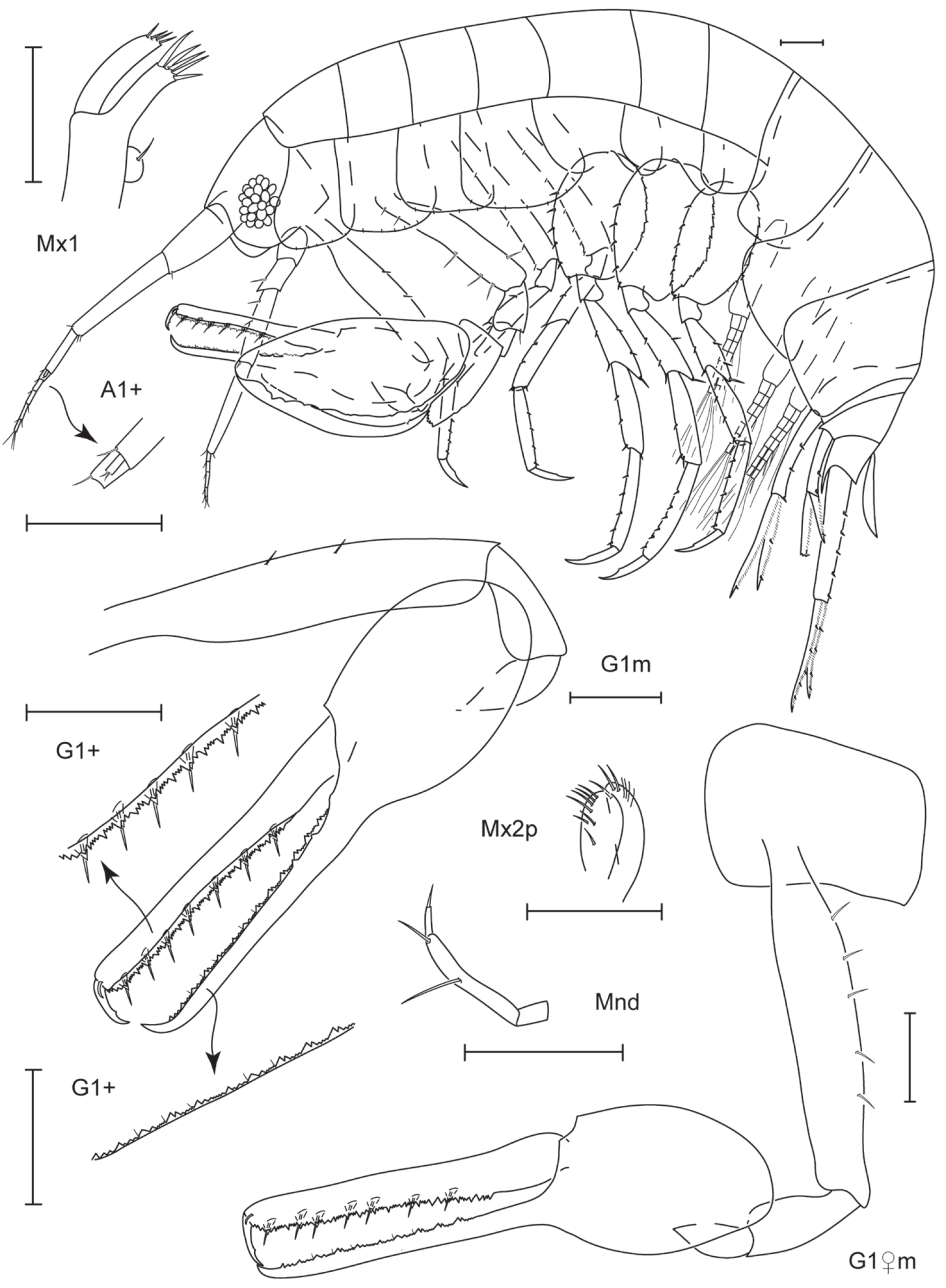
*Leucothoe hashi* sp. n., holotype male, 2.5 mm, RUMF-ZC-1747; paratype female, 2.5 mm, RUMF-ZC-1748.

**Figure 8.**
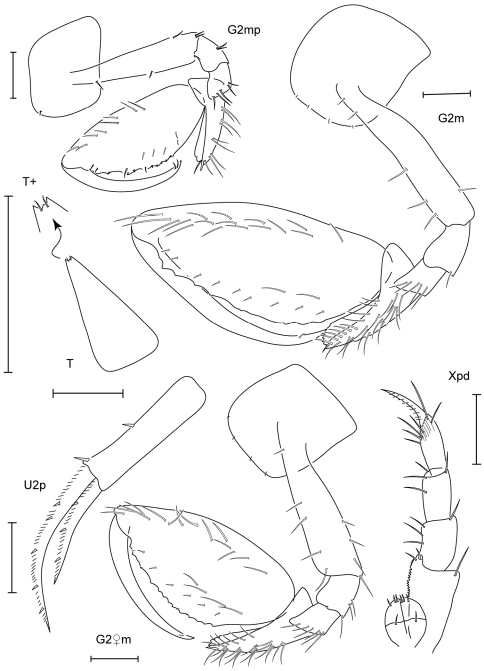
*Leucothoe hashi* sp. n., holotype male, 2.5 mm, RUMF-ZC-1747; paratype female, 2.5 mm, RUMF-ZC-1748; paratype male, 1.9 mm, RUMF-ZC-1749.

##### Etymology.

After the Japanese word ‘hashi’, meaning ‘chopsticks’ and referring to the extremely slender carpus and propodus of gnathopod 1. (Pronounced hah-shee.)

**Ecology.** In canals of sponges, *Callyspongia* sp., RUMF-ZP-2, KNWOkinawa42C (Figure 24F), ?*Clathria (Thalysias) reinwardti*, RUMF-ZP-4, 21Aug10 (Figure 25F), *Tedania* sp. (Figure 25E), *Haliclona* of Grant 1836, RUMF-ZP-3, KNWOkinawa44A (Figure 24G); and among coral rubble.

##### Relationships.

*Leucothoe hashi* sp. n. is similar to *Leucothoe cheiriserra* Serejo, 1998, *Leucothoe gavialis* Myers, 1985, *Leucothoe hipposideros* White & Thomas, 2009, *Leucothoe squalidens* Ledoyer, 1984, and *Leucothoe lecroyae* sp. n. in having triangular teeth on the palm of gnathopod 1 propodus. It also shares a rounded anterodistal head margin and distally truncate gnathopod 2 carpus with *Leucothoe hipposideros* and *Leucothoe squalidens*, but differs in having narrow pereopod 5–7 bases, a 1–articulate maxilla 1 palp, tuberculate maxilliped inner plate, and a 1–articulate accessory flagellum on antenna 1.

##### Remarks.

*Leucothoe hashi* sp. n. is translucent pink in color, darkest along pereonite edges (Figure 24A). This species has been collected from only 5 islands throughout the Ryukyu Archipelago. There appears to be some minor variation among specimens in the following characters: gnathopod 1 carpus dentition, propodus palm serration; gnathopod 2 shape and setal patterns.

##### Distribution.

East China Sea: Iriomote–jima Island, Okinawa–jima Island (both Okinawa), Tokunoshima Island, Amami–oshima Island, Yakushima Island (all Kagoshima), Japan.

#### Leucothoe lecroyae sp. n.

urn:lsid:zoobank.org:act:5089CA73-CBB4-4A13-A3A2-9F1CE93749FB

http://species-id.net/wiki/Leucothoe_lecroyae

Figures 9 10

##### Type material.

Holotype male, 3.5 mm RUMF-ZC-1763, Kuse, Kakeroma–jima Island reef wall (28°06'05"N, 129°21'12"E), in canals of brown sponge, *Rhabdastrella* of Thiele 1903, 10 m, K.N. White, col., 19 March 2011 (KNWAmami2C). Paratype female, 3.3 mm RUMF-ZC-1764, same station data as holotype.

##### Type locality.

Kuse, Kakeroma–jima Island, Amami–oshima Island region, Kagoshima, Japan (28°06'05"N, 129°21'12"E).

##### Additional material examined.

7 specimens, NSMT-Cr 21894, KNWYaku3D; 7 specimens, RUMF-ZC-1765, KNWYaku3D; 1 specimen, RUMF-ZC-1766, KNWYaku5P; 3 specimens, NSMT-Cr 21895 KNWOkinawa44A; 1 specimen, RUMF-ZC-1767, KNWOkinawa44F; 5 specimens, NSMT-Cr 21893, KNWAmami2D; 1 male specimen, RUMF-ZC-1799, KNWAmami2C.

##### Diagnosis (male).

Ventral cephalic keel anterior margin transverse, anteroventral margin quadrate. Eyes oval. Antennae 1 accessory flagellum 1–articulate. Right mandible with small 2–layered lacinia mobilis. Maxilliped inner plates small with serrate robust setae; outer plate tuberculate. Gnathopod 1 coxa anterior margin serrate; carpus robust, proximal margin serrate; propodus palm serrate with triangular teeth. Gnathopod 2 carpus distally truncate; propodus with 2 mediofacial setal rows. Pereopods 5–7 coxae with facial setae; bases posteriorly serrate. Female ventral cephalic keel anteroventral margin serrate; gnathopod 1 basis posterior margin with 6 short setae; gnathopod 2 carpus distally tapered.

##### Description (male).

Head. Anterior margin rounded, anterodistal margin evenly rounded; ventral cephalic keel anterior margin transverse, anteroventral margin quadrate, ventral margin oblique; eyes with more than 10 ommatidia, oval. Antenna 1 0.4 × body length, flagellum 10+–articulate (broken), peduncle article 1 width less than 2 × article 2, accessory flagellum 1–articulate. Antenna 2 0.3 × body length, slightly shorter than antenna 1, flagellum 6–articulate. Mandibular palp ratio of articles 1–3 1.0: 2.8: 1.0, article 2 with 2 medium distal setae, article 3 with 2 distal setae, incisors strongly dentate; left mandible with 8 raker spines, lacinia mobilis large, strongly toothed; right mandible with 7 raker spines, with small 2–layered lacinia mobilis, strongly dentate. Upper lip asymmetrically lobate, anterior margin setose. Lower lip inner lobes fused, setose; outer lobes with moderate gape, anterior margins setose. Maxilla 1 palp 1–articulate, margins constricted and with 4 distal setae; outer plate with 7 distal robust setae and 6 short marginal setae. Maxilla 2 inner plate with 4 robust distal setae, 2 robust facial setae, and 7 slender marginal setae; outer plate with 3 robust distal setae and 3 slender distal marginal setae. Maxilliped inner plates distal margin with a v-shaped indentation, with short simple and serrate robust setae; outer plate inner margin tuberculate, reaching 0.3 × palp article 1, with simple marginal setae, facial setae absent; palp article 4 subequal in length with article 3, distally acute.

Pereon. Coxae 1–4 relative widths 1.0: 1.5: 1.2: 1.8. Gnathopod 1 coxa smooth, with tiny marginal setae, anterior margin serrate, anterior margin produced, rounded, distal margin straight, posterior margin excavate, facial setae absent; basis linear, anterior margin with 1 short seta, posterior margin with 2 short setae; ischium bare; carpus linear, distal length 7.1 × width, proximal margin serrate, distal margin bare; propodus straight, palm serrate with 10 distal setae and 10 distal triangular teeth; dactylus smooth, reaching 0.1 × propodus length. Gnathopod 2 coxa broader than long, subequal in size with coxa 3, smooth, with tiny marginal setae, anterior margin expanded, anterodistally rounded, distal and posterior margins straight, facial setae absent; basis distally expanded, anterior margin with 10 medium setae, posterior margin with 2 short setae; ischium with 1 posterodistal seta; carpus 0.4 × propodus length, curved, distally truncate, anterior margin dentate; propodus with 2 mediofacial setal rows, primary mediofacial setal row above midline, reaching 0.8 × propodus length, secondary mediofacial setal row with 4 setae, with 1 row of submarginal setae, posterior margin with 5 small robust setae, palm convex with 10 tubercles; dactylus curved, proximal margin smooth with 1 seta, anterior margin distally subacute, reaching 0.5 × propodus length. Pereopod 3 coxa length 1.3 × width, anterodistal corner overriding distal face of coxa 2 and extending below it, smooth, with tiny marginal setae, anterior margin straight, distal margin oblique, posterior margin straight, facial setae absent. Pereopod 4 coxa smooth, with tiny marginal setae, anterior margin produced, distal margin evenly rounded, posterior margin tapered, facial setae absent. Pereopods 5–7 coxae facial setae present; bases width length ratios 1: 1.4, 1: 1.3, 1: 1.2, serrate, setose.

Pleon. Epimera 1–2 with ventral setae, epimeron 3 bare; epimeron 3 posteroventral corner sinuous, rounded. Uropods 1–3 relative lengths 1.0: 0.8: 0.9. Uropod 1 peduncle subequal in length with inner ramus, outer ramus 0.9 × inner ramus length; inner ramus with 6 robust setae and outer ramus with 4 robust setae. Uropod 2 peduncle 0.9 × inner ramus length, outer ramus length 0.6 × inner ramus length; inner ramus with 4 robust setae and outer ramus with 3 robust setae. Uropod 3 peduncle 1.2 × inner ramus length, outer ramus length 0.9 × inner ramus length; inner ramus with 3 robust setae and outer ramus with 5 robust setae. Telson 2.3 × longer than wide, apex strongly tridentate.

**Female (sexually dimorphic characters).**

Ventral cephalic keel anteroventral margin serrate. Gnathopod 1 basis posterior margin with 6 short setae. Gnathopod 2 basis posterior margin with 1 seta; ischium with 2 distal and 2 posterodistal setae; carpus distally tapered, anterior margin smooth; propodus posterior margin bare.

**Figure 9.**
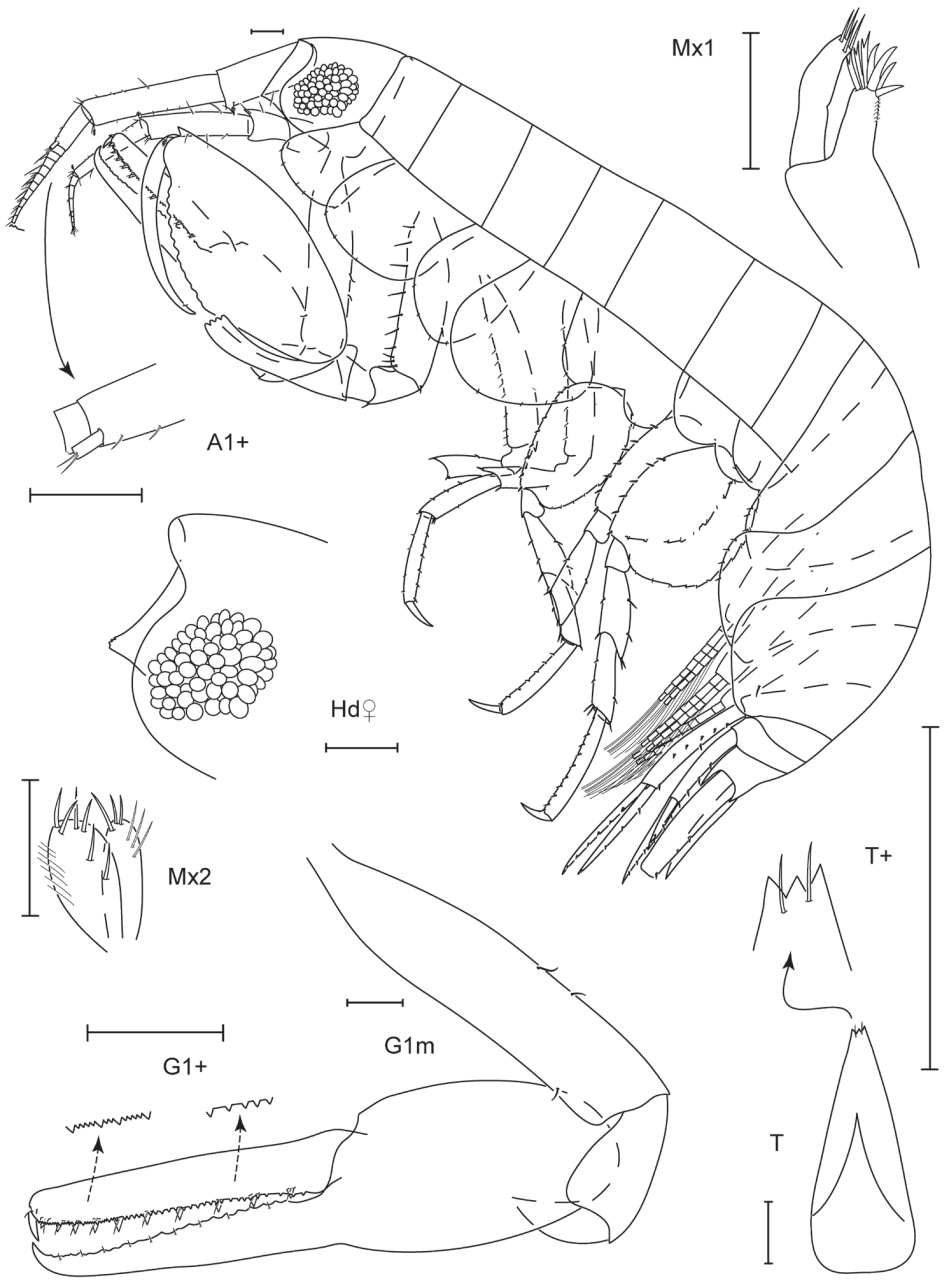
*Leucothoe lecroyae* sp. n., holotype male, 3.5 mm, RUMF-ZC-1763; paratype female, 3.3 mm, RUMF-ZC-1764.

**Figure 10.**
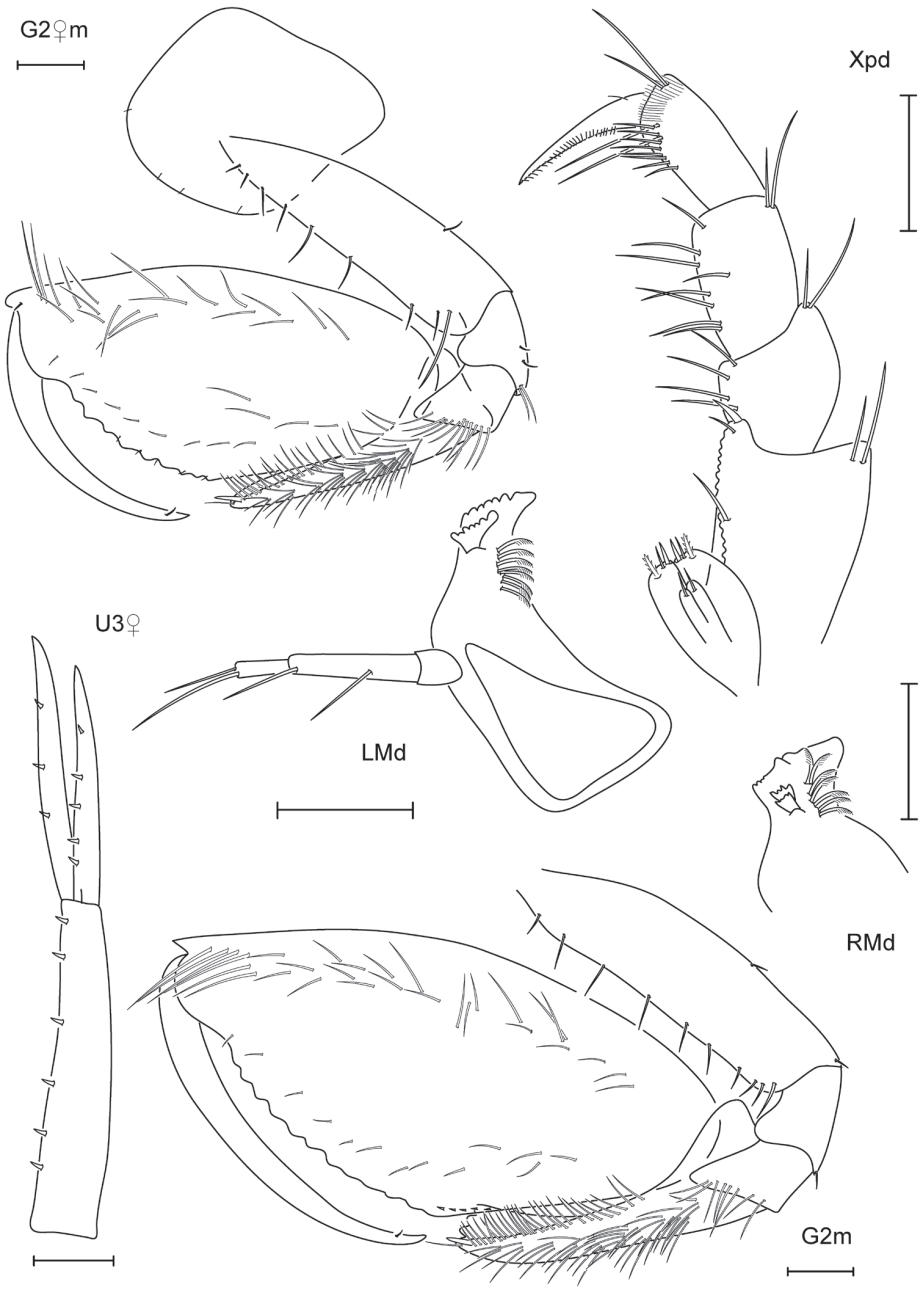
*Leucothoe lecroyae* sp. n., holotype male, 3.5 mm, RUMF-ZC-1763; paratype female, 3.3 mm, RUMF-ZC-1764.

##### Etymology.

Named for Sara E. LeCroy, in recognition of her contribution to amphipod taxonomy. Ms LeCroy has been a colleague and friend for the past 7 years and the first author is very grateful for all her support.

##### Ecology.

In canals of sponges, brown sponge with holes on top only, *Rhadastrella* sp., RUMF-ZP-1, KNWOkinawa16A (Figure 24H), dark red chimney sponge, Axinellidaeof Carter 1875, RUMF-ZP-12, KNWYaku3F (Figure 24D), orange lumpy sponge, Axinellidae (Figure 24J); and among coral rubble.

##### Relationships.

*Leucothoe lecroyae* sp. n. is similar to *Leucothoe cheiriserra* Serejo, 1998, *Leucothoe gavialis* Myers, 1985, *Leucothoe hipposideros* White & Thomas, 2009, *Leucothoe squalidens* Ledoyer, 1984, and *Leucothoe hashi* sp. n. in having triangular teeth on the palm of gnathopod 1 propodus. It also shares a short dactylus on gnathopod 1, rounded head margin, and distally truncate gnathopod 2 carpus with *Leucothoe hashi* sp. n., but differs in having a more robust gnathopod 1 propodus and carpus, maxilla 1 palp 1–articulate, margins constricted, 2–layered lacinia mobilis on the right mandible, and wide pereopod 5–7 bases.

##### Remarks.

*Leucothoe lecroyae* sp. n. is faint yellow in color (Figure 23C). This species has been collected on Yakushima Island and Amami–oshima Island region (both Kagoshima) and from the northwestern and eastern coasts of Okinawa–jima Island, Okinawa, Japan.

##### Distribution.

East China Sea: Okinawa–jima Island (Okinawa), Amami–oshima Island, Yakushima Island (both Kagoshima), Japan.

#### Leucothoe nagatekubi sp. n.

urn:lsid:zoobank.org:act:5416F771-FBE3-4D5F-A7A2-2D25A582ADA0

http://species-id.net/wiki/Leucothoe_nagatekubi

Figures 11 12

##### Type material.

Holotype male, 3.0 mm RUMF-ZC-1768, Mizugama, Okinawa–jima Island, Okinawa, reef wall (26°21'35"N, 127°44'22"E), in canals of orange encrusting sponge, *Clathria* of Schmidt 1862, 10 m, N.S. White, col., 26 February 2011 (KNWOkinawa34K). Paratype female, 3.4 mm RUMF-ZC-1769, Mizugama, Okinawa reef wall (26°21'35"N, 127°44'22"E), in canals of orange encrusting sponge, *Clathria*, 7 m, N.S. White, col., 20 October 2011 (KNWOkinawa69D)

##### Type locality.

Mizugama, Okinawa–jima Island, Okinawa, Japan (26°21'35"N, 127°44'22"E).

##### Additional material examined.

9 specimens, NSMT-Cr 21896, KNWOkinawa69B; 9 specimens, RUMF-ZC-1770, KNWOkinawa69C.

##### Diagnosis (male).

Head anterior margin truncate, anterodistal margin quadrate with cusp. Maxilla 1 palp 1–articulate. Maxilliped outer plate tuberculate with facial setae. Gnathopod 1 basis anterodistal margin with 7 very short, slender setae. Gnathopod 2 carpus elongate, distally tapered; propodus palm with large concavity. Pereopod 7 basis posteriorly serrate. Telson with plumose facial setae.

##### Description (male).

Head. Anterior margin truncate, anterodistal margin quadrate with cusp; ventral cephalic keel anterior margin transverse, anteroventral margin quadrate, ventral margin distally excavate, rounded; eyes with more than 10 ommatidia, round. Antenna 1 0.3 × body length, flagellum 7–articulate, peduncle article 1 width less than 2 × article 2, accessory flagellum 1–articulate. Antenna 2 0.3 × body length, subequal in length with antenna 1, flagellum 4–articulate. Mandibular palp ratio of articles 1–3 1.0: 3.0: 1.7, article 2 with 2 medium distal setae, article 3 with 2 distal setae, incisors strongly dentate; left mandible with 5 raker spines, lacinia mobilis large, strongly toothed; right mandible with 7 raker spines, lacinia mobilis small, strongly dentate. Upper lip asymmetrically lobate, anterior margin setose. Lower lip inner lobes fused, bare; outer lobes with moderate gape, anterior margins setose. Maxilla 1 palp 1–articulate with 4 distal setae; outer plate with 5 distal robust setae. Maxilla 2 inner plate with 3 robust distal setae and 7 slender marginal setae; outer plate with 3 robust distal setae and 15 slender distal marginal setae. Maxilliped inner plates distal margin with a v-shaped indentation, with short robust setae; outer plate inner margin tuberculate, reaching 0.1 × palp article 1, with simple marginal setae, facial setae present; palp article 4 subequal in length with article 3, distally acute.

Pereon. Coxae 1–4 relative widths 1.0: 1.8: 1.1: 2.1. Gnathopod 1 coxa smooth, with tiny marginal setae, anterodistal margin produced, rounded with cusp, distal margin straight, posterior margin excavate, facial setae absent; basis linear, anterodistal margin with 7 very short, slender setae, posterior margin bare; ischium bare; carpus linear, distal length 13.1 × width, proximal margin serrate, distal margin with 1 short seta; propodus straight, palm with 6 distal triangular teeth and 6 distal setae; dactylus smooth, reaching 0.1 × propodus length. Gnathopod 2 coxa broader than long, subequal in size with coxa 3, smooth, with tiny marginal setae, anterior margin expanded anteriorly, anterodistally rounded, distal margin oblique, posterior margin straight, facial setae absent; basis distally expanded, anterior margin with 5 medium setae, distal margin with 2 setae, posterior margin with 3 setae; ischium with 3 posterior, 2 distal, and 3 posterodistal setae; carpus 0.5 × propodus length, curved, distally tapered, anterior margin smooth; propodus with 1 mediofacial setal row above midline, reaching 0.8 × propodus length, with 2 submarginal setae, posterior margin smooth, palm convex with 6 major tubercles and 1 major concavity; dactylus curved, proximal margin smooth with 1 seta, anterior margin distally acute, reaching 0.5 × propodus length. Pereopod 3 coxa length 1.5 × width, anterodistal corner overriding distal face of coxa 2 and extending below it, smooth, with tiny marginal setae, anterior margin evenly rounded, distal margin oblique, posterior margin straight, facial setae absent. Pereopod 4 coxa smooth, with tiny marginal setae, anterior margin produced, distal margin evenly rounded, posterior margin tapered, facial setae absent. Pereopods 5–7 coxae facial setae absent; bases width length ratios 1: 1.3, 1: 1.3, 1: 1.2, posterior margins setose. Pereopods 5–6 bases posterior margins smooth, pereopod 7 basis posterior margin serrate.

Pleon. Epimera 1 and 3 bare, epimeron 2 with ventral setae; epimeron 3 posteroventral corner rounded. Uropods 1–3 relative lengths 1.0: 0.7: 0.9. Uropod 1 peduncle and outer ramus subequal in length with inner ramus; inner ramus with 5 robust setae and outer ramus with 2 robust setae. Uropod 2 peduncle 0.9 × inner ramus length, outer ramus 0.7 × inner ramus length; inner ramus with 1 robust seta and outer ramus with 2 robust setae. Uropod 3 peduncle 1.6 × inner ramus length, outer ramus 0.9 × inner ramus length; inner ramus with 1 robust seta and outer ramus with 2 robust setae. Telson 2.3 × longer than wide, with plumose facial setae, apex strongly tridentate.

**Female (sexually dimorphic characters).**

Unknown.

**Figure 11.**
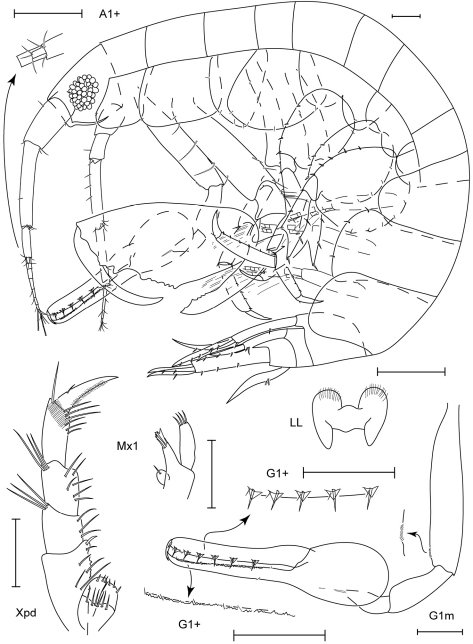
*Leucothoe nagatekubi* sp. n., holotype male, 3.0 mm, RUMF-ZC-1768.

**Figure 12.**
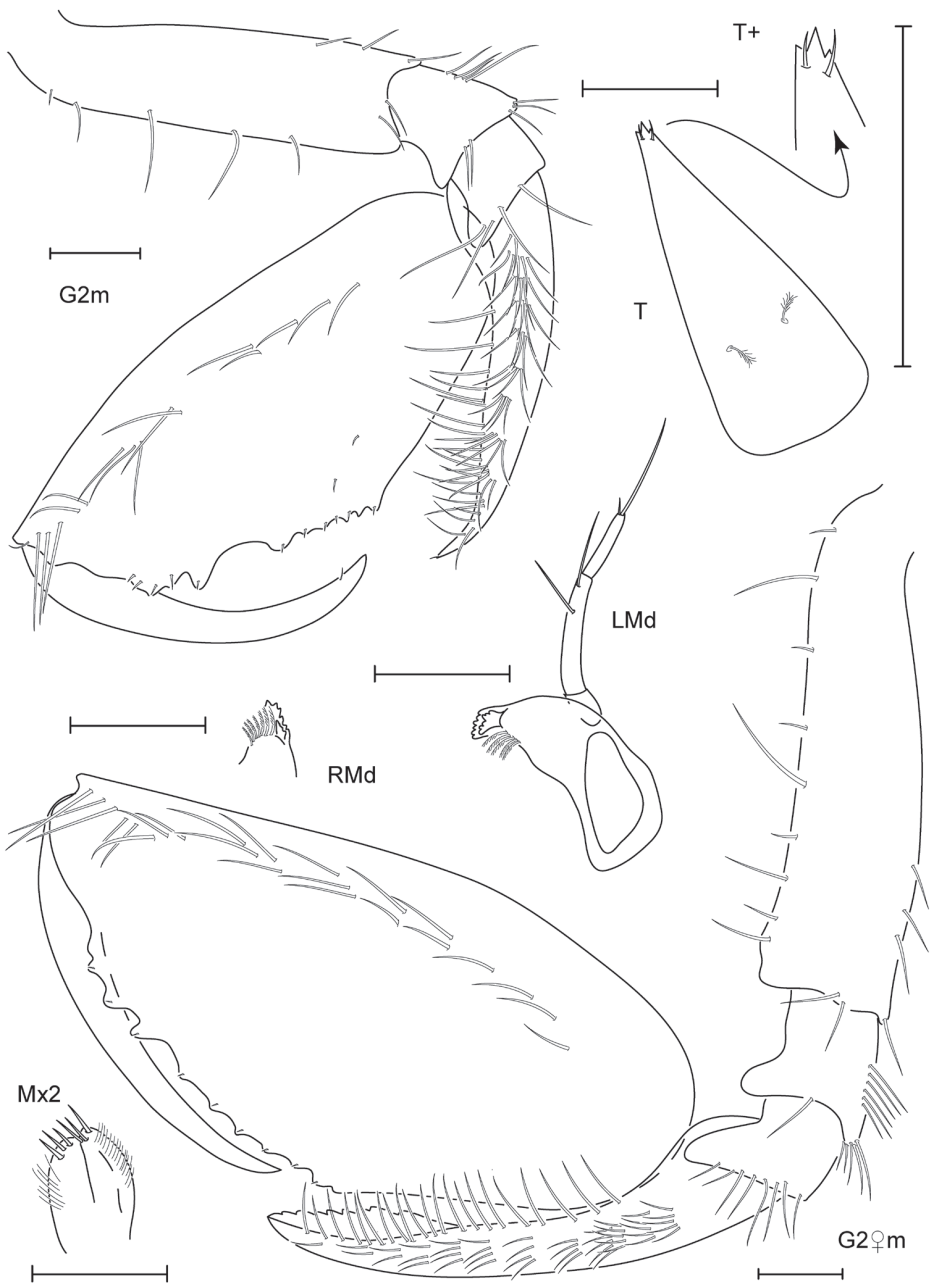
*Leucothoe nagatekubi* sp. n., holotype male, 3.0 mm, RUMF-ZC-1768; paratype female, 3.4 mm, RUMF-ZC-1769.

##### Etymology.

After the Japanese words ‘nagai’, meaning ‘long’, and ‘tekubi’, meaning ‘wrist’ and referring to the elongate carpus on gnathopod 2. (Pronounced na-ga-tay-koo-bee)

##### Ecology.

In canals of orange encrusting sponge, *Clathria* sp., RUMF-ZP-5, KNWOkianawa69G (Figure 25D), found in caves.

##### Relationships.

*Leucothoe nagatekubi* sp. n. is similar to *Leucothoe serrata* White & Thomas, 2009 in having an elongate gnathopod 2 carpus, narrow gnathopod 1 propodus and carpus, high mediofacial setal row on gnathopod 2 propodus, and wide pereopod 5–7 bases. It differs in the large indentation on the gnathopod 2 propodus palm, having a 1–articulate accessory flagellum on antenna 1, triangular teeth on gnathopod 1 propodus palm, and a 1–articulate maxilla 1 palp.

##### Remarks.

*Leucothoe nagatekubi* sp. n. is pinkish-orange in color (Figure 24C). This species is endemic to the western coast of Okinawa–jima Island, Okinawa.

##### Distribution.

East China Sea: Okinawa–jima Island, Okinawa, Japan.

#### Leucothoe nurunuru sp. n.

urn:lsid:zoobank.org:act:02C845F0-D0F4-4E56-AED4-0970D1310F53

http://species-id.net/wiki/Leucothoe_nurunuru

Figures 13 14

##### Type material.

Holotype male, 5.8 mm RUMF-ZC-1771, Channel between Iriomote–jima Island and Hatoma–jima Island, Okinawa, patch reef (24°26'34"N, 123°49'18"E), in canals of slimy black-purple sponge, Iotrochotidae of Dendy 1922 (probably *Iotrochota* of Ridley 1884), 10 m, K.N. White and N.S. White, col., 22 April 2011 (KNWIriomote2F). Paratype female, 6.5 mm RUMF-ZC-1772, same station data as holotype.

##### Type locality.

Iriomote Channel between Iriomote–jima and Hatoma–jima Islands (24°26'34"N, 123°49'18"E).

##### Additional material examined.

4 specimens, RUMF-ZC-1773, KNWIriomote2F; 4 specimens, NSMT-Cr 21897, KNWIriomote2F.

##### Diagnosis (male).

Antenna 1 accessory flagellum 1–articulate. Maxilla 1 palp 1–articulate, margins constricted. Maxilliped outer plate with setulate-serrate marginal setae. Gnathopod 1 coxa with 2 mediofacial setae; propodus palm with square-shaped denticles. Gnathopod 2 carpus distally truncate; propodus with long submarginal setae. Pereopod 5 coxa with 2 facial setae. Telson apex with strongly rounded point. Female gnathopod 1 basis anterior margin with 16 short setae, posterior margin with 14 short setae; gnathopod 2 basis anterior margin with 14 short and 2 long curved setae.

##### Description (male).

Head, anterior margin rounded, anterodistal margin evenly rounded; ventral cephalic keel anterior margin excavate, anteroventral margin subquadrate, ventral margin oblique; eyes with more than 10 ommatidia, round. Antenna 1 0.3 × body length, flagellum 11–articulate, peduncle article 1 width less than 2 × article 2, accessory flagellum 1–articulate. Antenna 2 0.3 × body length, subequal in length with antenna 1, flagellum 7–articulate. Mandibular palp ratio of articles 1–3 1.0: 2.6: 1.1, article 2 with 6–8 long distal setae, article 3 with 2 distal setae, incisors strongly dentate; left mandible with 12 raker spines, lacinia mobilis large, strongly toothed; right mandible with 13 raker spines, lacinia mobilis small, strongly dentate. Upper lip asymmetrically lobate, anterior margin setose. Lower lip inner lobes fused, setose; outer lobes with moderate gape, anterior margins setose. Maxilla 1 palp 1–articulate, margins constricted and with 3 distal setae; outer plate with 7 distal robust setae. Maxilla 2 inner plate with 6 robust distal setae and several slender facial setae; outer plate with 4 robust distal setae and 5 slender distal setae. Maxilliped inner plates distal margin with a v-shaped indentation, with short robust setae; outer plate inner margin smooth, reaching 0.2 × palp article 1, with simple and setulate-serrate marginal setae, facial setae absent; palp article 4 subequal in length with article 3, distally acute.

Pereon. Coxae 1–4 relative widths 1.0: 1.0: 0.8: 1.4. Gnathopod 1 coxa smooth, with tiny marginal setae, anterodistal margin produced, subquadrate, distal margin straight, posterior margin excavate, 2 mediofacial setae present; basis proximally widened, anterior margin with 7 short setae, posterior margin with 6 short setae; ischium bare; carpus linear, distal length 13.1 × width, proximal margin smooth, distal margin bare; propodus straight, palm with square-shaped denticles with 6 large and 20 small proximal setae; dactylus smooth, with 2 distal setae, reaching 0.5 × propodus length. Gnathopod 2 coxa as long as broad, subequal in size with coxa 3, smooth, with tiny marginal setae, anterior margin expanded anteriorly, anterodistally rounded, distal and posterior margins rounded, facial setae absent; basis slightly posteriorly expanded, anterior margin with 7 short and 2 long curved setae, posterior margin with 1 posterodistal seta, distal margin with 2 setae; ischium with 3 short posterior, 2 long distal, and 5 short posterodistal setae; carpus 0.4 × propodus length, curved, distally truncate, anterior margin smooth; propodus with 1 mediofacial setal row displaced to midline, reaching 0.8 × propodus length, with 1 row of short and long submarginal setae, posterior margin smooth, palmar corner pronounced, palm convex with small tubercles; dactylus curved, proximal margin smooth with 2 setae, anterior margin distally acute, reaching 0.6 × propodus length. Pereopod 3 coxa length 1.4 × width, anterodistal corner overriding distal face of coxa 2 and extending below it, smooth, with tiny marginal setae, anterior margin expanded, distal margin slightly convex, posterior margin tapered, facial setae absent. Pereopod 4 coxa smooth, with tiny marginal setae, anterior margin produced, distal margin evenly rounded, posterior margin excavate, facial setae absent. Pereopod 5 coxa with 2 facial setae. Pereopods 6–7 coxae facial setae absent. Pereopods 5–7 bases width length ratios 1: 1.2, 1: 1.2, 1: 1.0, posterior margins smooth, setose.

Pleon. Epimera 1–2 with ventral setae, epimeron 3 bare; epimeron 3 posteroventral corner subquadrate. Uropods 1–3 relative lengths 1.0: 0.7: 1.0. Uropod 1 peduncle 0.8 × inner ramus length, outer ramus 0.9 × inner ramus length; inner ramus with 4 robust setae and outer ramus with 6 robust setae. Uropod 2 peduncle subequal in length with inner ramus, outer ramus 0.8 × inner ramus length; inner ramus with 5 robust setae and outer ramus with 3 robust setae. Uropod 3 peduncle 1.2 × inner ramus length, outer ramus 0.8 × inner ramus length; inner and outer rami lined with short marginal setae; inner ramus with 4 robust setae and outer ramus with 5 robust setae. Telson 2.3 × longer than wide, apex with strongly rounded point.

**Female (sexually dimorphic characters).**

Gnathopod 1 basis anterior margin with 16 short setae, posterior margin with 14 short setae; ischium with 3 short posterior setae. Gnathopod 2 basis anterior margin with 14 short and 2 long curved setae; ischium with 3 distal and 6 posterodistal setae; carpus slightly less truncate than in the male.

**Figure 13.**
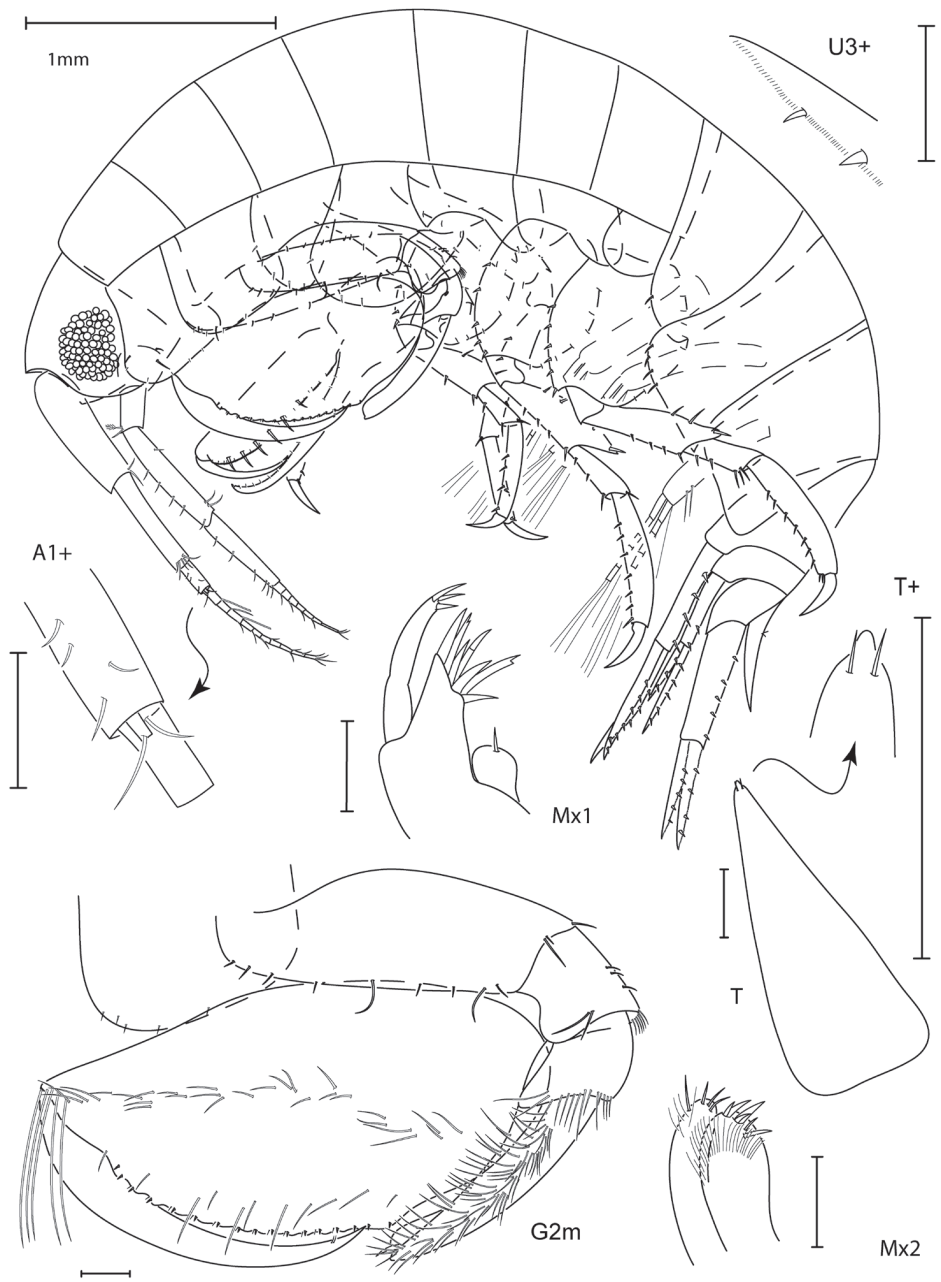
*Leucothoe nurunuru* sp. n., holotype male, 5.8 mm, RUMF-ZC-1771.

**Figure 14.**
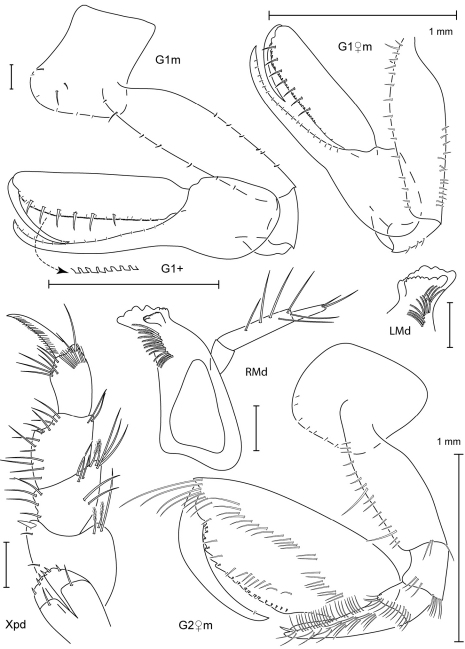
*Leucothoe nurunuru* sp. n., holotype male, 5.8 mm, RUMF-ZC-1771; paratype female, 6.5 mm, RUMF-ZC-1772.

##### Etymology.

After the Japanese word ‘nurunuru’, meaning ‘slimy’ and referring to the host sponge. (Pronounced new-rue-new-rue)

##### Ecology.

In canals of Iotrochotidae (probably *Iotrochota* sp.), RUMF-ZP-7, KNWIriomote2G (Figure 25A).

##### Relationships.

*Leucothoe nurunuru* sp. n.is similar to *Leucothoe commensalis* (Haswell, 1879), *Leucothoe procera* (Bate, 1857), *Leucothoe makromattos* White & Thomas, 2009, *Leucothoe daisukei* sp. n., and *Leucothoe akaoni* sp. n. in having a round anterior head margin and long gnathopod 1 dactylus. The pointed apex of the telson is similar to *Leucothoe commensalis* and *Leucothoe procera*, although the point is much stronger in *Leucothoe nurunuru* sp. n. *Leucothoe nurunuru* sp. n. differs from these two species in having maxilla 1 palp 1–articulate, margins constricted, wider pereopod 5–7 bases, and epimeron 3 posteroventral corner subquadrate. *Leucothoe nurunuru* sp. n. also shares wide pereopod 5–7 bases and a setose posterior margin of gnathopod 1 basis with *Leucothoe makromattos*, *Leucothoe daisukei* sp. n., and *Leucothoe akaoni* sp. n., but differs in having maxilla 1 palp 1–articulate, margins constricted and square-shaped denticles on gnathopod 1 propodus palm.

##### Remarks.

*Leucothoe nurunuru* sp. n. is deep orange in color (Figure 23D). This species is endemic to Iriomote–jima Island.

##### Distribution.

East China Sea: Iriomote–jima Island, Okinawa, Japan.

#### Leucothoe ouraensis sp. n.

urn:lsid:zoobank.org:act:6A8C5B7D-3111-4AEE-AEFB-0F3A26FE01A4

http://species-id.net/wiki/Leucothoe_ouraensis

Figures 15 16

##### Type material.

Holotype male, 3.3 mm RUMF-ZC-1774, Kita–nakase, Oura–wan Bay, Okinawa–jima Island, Okinawa, patch reef (26°32'40"N, 128°03'36"E), among coral rubble, 30 m, K.N. White and N.S. White, col., 3 March 2011 (KNWOkinawa35F). Paratype female, 3.0 mm RUMF-ZC-1775, Tettou–mae–oki, Oura–wan Bay, Okinawa–jima Island, Okinawa, patch reef (26°32'43"N, 128°02'56"E), among coral rubble, 28 m, K.N. White, col., 4 April 2011 (KNWOkinawa37G).

##### Type locality.

Oura–wan Bay, Okinawa–jima Island, Okinawa, Japan (26°32'40–43"N, 128°02'56"–03'36"E).

##### Additional material examined.

1 specimen, RUMF-ZC-1776, KNWOkinawa39H; 1 specimen, NSMT-Cr 21898, KNWOkinawa39L; 1 specimen, RUMF-ZC-1777, KNWOkinawa37C; 2 specimens, NSMT-Cr 21899, KNWOkinawa37N.

##### Diagnosis (male).

Head anterior margin truncate, anterodistal margin quadrate. Ventral cephalic keel anteroventral margin with anteriorly projecting cusp. Right mandible lacinia mobilis cleft. Upper lip distal margin with row of short setae. Maxilla 1 palp 1–articulate, proximal margin serrate. Maxilliped outer plate tuberculate. Gnathopod 1 basis posterior margin with 6 short setae; propodus palm serrate; dactylus reaching 0.1 × propodus length. Gnathopod 2 carpus distally truncate; propodus with 2 mediofacial setal rows. Uropod 3 peduncle 1.2 × inner ramus length. Telson apex bidentate.

##### Description (male).

Head. Anterior margin truncate, anterodistal margin quadrate; ventral cephalic keel anterior margin oblique, anteroventral margin with anteriorly projecting cusp, ventral margin convex; eyes with more than 10 ommatidia, round. Antenna 1 0.3 × body length, flagellum 8–articulate, peduncle article 1 width less than 2 × article 2, accessory flagellum absent. Antenna 2 0.3 × body length, subequal in length with antenna 1, flagellum 5–articulate. Mandibular palp ratio of articles 1–3 1.0: 2.4: 1.6, article 2 with 3 medium distal setae, article 3 with 2 distal setae, incisors weakly dentate; left mandible with 9 raker spines, lacinia mobilis large, strongly toothed; right mandible with 7 raker spines, lacinia mobilis small, strongly dentate, cleft. Upper lip asymmetrically lobate, anterior margin setose, distal margin with row of short setae. Lower lip inner lobes fused, setose; outer lobes with moderate gape, anterior margins setose. Maxilla 1 palp 1–articulate with 4 distal setae, proximal margin serrate; outer plate with 4 distal robust setae and 4 distal setae. Maxilla 2 inner plate with 3 robust distal setae and 4 slender marginal setae; outer plate with 2 robust and 2 slender distal setae and 6 slender distal marginal setae. Maxilliped inner plates distal margin with a v-shaped indentation, with short robust setae; outer plate inner margin tuberculate, reaching 0.1 × palp article 1, with simple marginal setae, facial setae present; palp article 4 subequal in length with article 3, distally acute.

Pereon. Coxae 1–4 relative widths 1.0: 1.2: 0.9: 1.7. Gnathopod 1 coxa smooth, with tiny marginal setae, anterior margin produced, rounded, distal margin straight, posterior margin excavate, facial setae absent; basis distally expanded, anterior margin bare, posterior margin with 6 short setae; ischium bare; carpus linear, distal length 13.6 × width, proximal margin smooth, distal margin bare; propodus straight, palm serrate with 9 distal setae; dactylus smooth, reaching 0.1 × propodus length. Gnathopod 2 coxa as long as broad, subequal in size with coxa 3, smooth, with tiny marginal setae, anterior margin straight, anterodistally rounded, distal margin evenly rounded, posterior margin straight, facial setae absent; basis distally expanded, anterior margin with 9 setae, posterior margin with 4 setae; ischium with 1 posterodistal seta; carpus 0.4 × propodus length, curved, distally truncate, anterior margin dentate; propodus with 2 mediofacial setal rows, primary mediofacial setal row above midline, reaching 0.8 × propodus length, secondary mediofacial setal row with 2 setae, with 1 row of submarginal setae, posterior margin smooth, palm convex, dentate; dactylus curved, proximal margin smooth with 1 seta, anterior margin distally acute, reaching 0.6 × propodus length. Pereopod 3 coxa length 1.4 × width, anterodistal corner overriding distal face of coxa 2 and extending below it, smooth, with tiny marginal setae, anterior margin straight, distal margin slightly convex, posterior margin straight, facial setae absent. Pereopod 4 coxa smooth, with tiny marginal setae, anterior margin produced, distal margin evenly rounded, posterior margin tapered, facial setae absent. Pereopods 5–7 coxae facial setae absent. Pereopods 5–7 bases width length ratios 1: 1.4, 1: 1.2, 1: 1.1; posterior margins smooth, setose.

Pleon. Epimera 1–2 with ventral setae, epimeron 3 bare; epimeron 3 posteroventral corner rounded. Uropods 1–3 relative lengths 1.0: 0.8: 1.1; outer rami lined with short marginal setae. Uropod 1 peduncle and outer ramus subequal in length with inner ramus; inner ramus with 2 robust setae; outer ramus without robust setae. Uropod 2 peduncle length 0.8 × inner ramus, outer ramus length 0.6 × inner ramus; inner ramus with 4 robust setae; outer ramus with 3 robust setae. Uropod 3 peduncle length 1.2 × inner ramus, outer ramus subequal in length with inner ramus; inner and outer rami lined with short marginal setae, each with 4 robust setae. Telson 2.7 × longer than wide, apex bidentate.

**Female (sexually dimorphic characters).**

Gnathopod 2 ischium with posterior and posterodistal setae; carpus distally tapered, anterior margin smooth; propodus secondary mediofacial setal row with 4 setae.

**Figure 15.**
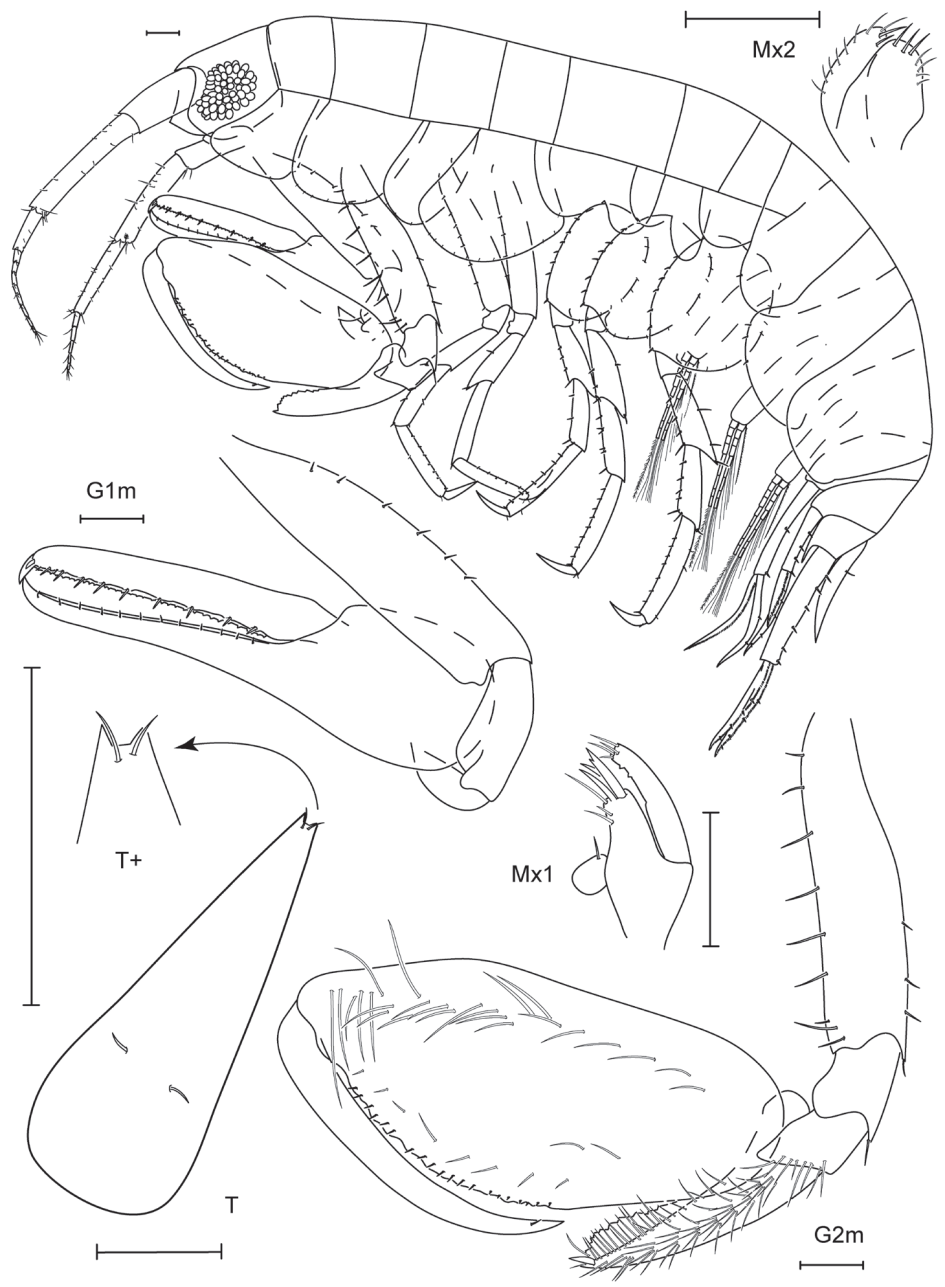
*Leucothoe ouraensis* sp. n., holotype male, 3.3 mm, RUMF-ZC-1774.

**Figure 16.**
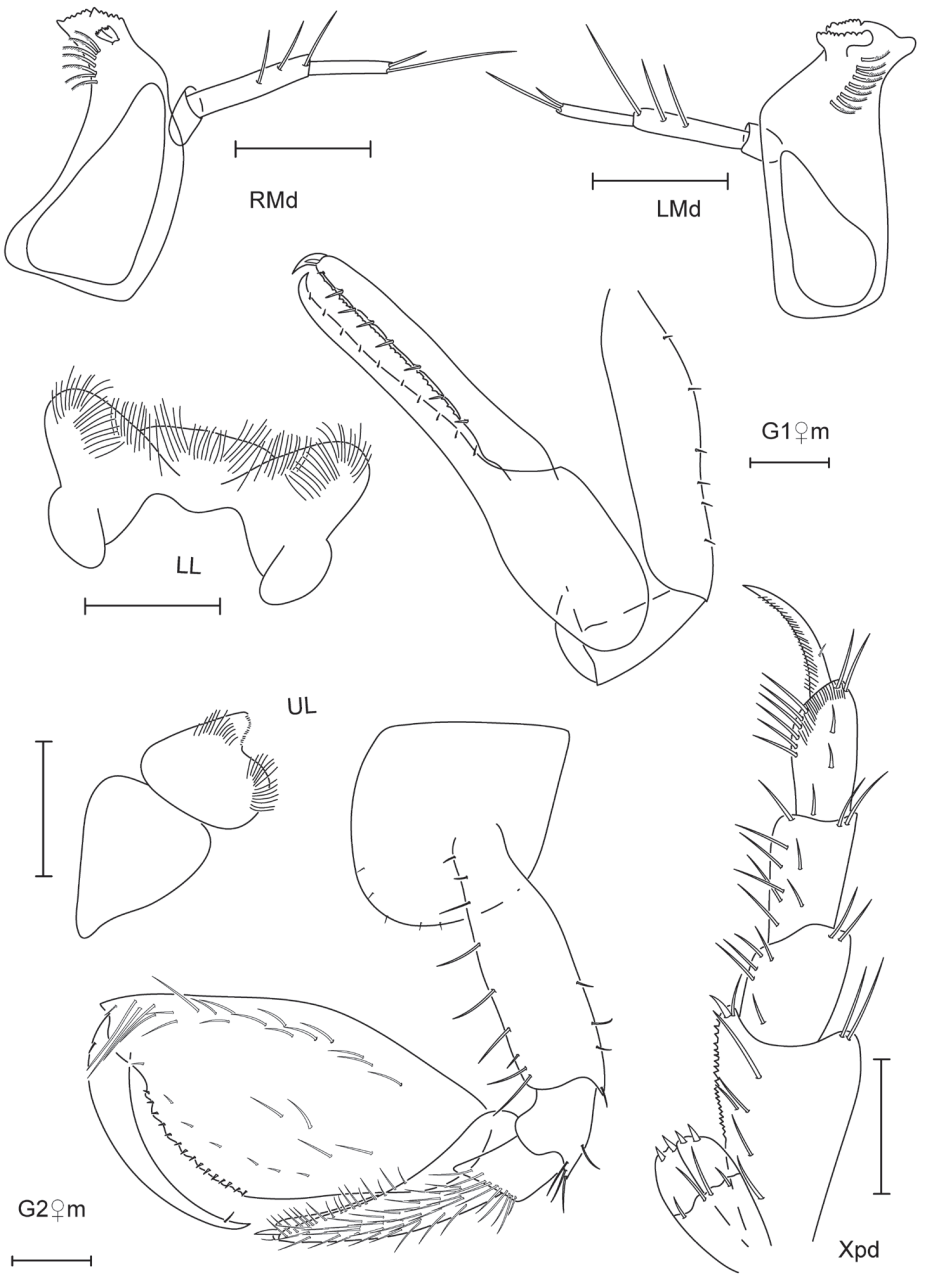
*Leucothoe ouraensis* sp. n., holotype male, 3.3 mm, RUMF-ZC-1774; paratype female, 3.0 mm, RUMF-ZC-1775.

##### Etymology.

After the Japanese place name ‘Oura’, meaning ‘large inlet’ and referring to the type locality. (Pronounced oh-ooh-ra)

**Ecology.** In canals of brown-red chimney sponge, *Mycale* of Gray 1867, RUMF-ZP-8, KNWOkinawa37K (Figure 25B), brown-red holey sponge, *Mycale (Zygomycale) parishii* (Bowerbank, 1875), RUMF-ZP-9, KNWOkinawa37N (Figure 25G), brown bivalve encrusting sponge, ?*Mycale* of Gray, 1867, RUMF-ZP-10, KNWOkinawa37M (Figure 25H); and among coral rubble.

##### Relationships.

*Leucothoe ouraensis* sp. n. is similar to *Leucothoe ctenochasma* Moore, 1987 in having gnathopod 1 dactylus reaching less than 0.1 × propodus length and a bidentate apex on the telson. This species differs from *Leucothoe ctenochasma* in having a truncate anterior head margin, quadrate anterodistal head margin, 1–articulate maxilla 1 palp, setose posterior margin on gnathopod 1 basis, and pereopods 5–7 bases broadly expanded and posteriorly smooth.

##### Remarks.

*Leucothoe ouraensis* sp. n. is yellow-orange in color (Figure 23E). This species is endemic to the eastern coast of Okinawa–jima Island, Okinawa.

##### Distribution.

East China Sea: Okinawa–jima Island, Okinawa, Japan.

#### Leucothoe togatta sp. n.

urn:lsid:zoobank.org:act:8846964A-A117-496F-B735-2B6039964F76

http://species-id.net/wiki/Leucothoe_togatta

Figures 17 18

##### Type material.

Holotype male, 4.2 mm RUMF-ZC-1778, Yoshida, Yakushima Island, Kagoshima, patch reef (30°23'58"N, 130°25'53"E), among coral rubble, 3–7 m, K.N. White and N.S. White, col., 26 May 2011 (KNWYaku2E). Paratype female, 5.4 mm RUMF-ZC-1779, same station data as holotype.

##### Type locality.

Yoshida, Yakushima Island, Kagoshima, Japan (30°23'58"N, 130°25'53"E).

##### Additional material examined.

2 specimens, RUMF-ZC-1780, KNWYaku5M; 1 specimen, NSMT-Cr 21900, KNWOkinawa35A; 1 specimen, RUMF-ZC-1781, KNWOkinawa36E; 1 specimen, RUMF-ZC-1782, KNWYaku1F; 1 specimen, NSMT-Cr 21901, KNWOkinawa51D; 1 specimen, RUMF-ZC-1783, KNWYaku1G; 1 specimen, KNWOkinawa55E.

##### Diagnosis (male).

Ventral cephalic keel anteroventral margin quadrate with large projection. Antenna 1 accessory flagellum 1–articulate. Mandibular palp article 2 with 11 long distal setae; article 2 with facial setae and 1 distal seta. Gnathopod 2 basis anterior margin with 7 long curved setae; carpus with setulate-serrate marginal setae; propodus with 3 mediofacial setal rows. Pereopod 7 basis posterior margin serrate. Female gnathopod 1 carpus elongate; gnathopod 2 basis anterior margin with 22 long curved setae, distal margin with 4 long curved setae; ischium with 3 long and 2 short distal setae, carpus distally truncate.

##### Description (male).

Head. Anterior margin rounded, anterodistal margin evenly rounded; ventral cephalic keel anterior margin excavate, anteroventral margin quadrate with projection, ventral margin convex; eyes with more than 10 ommatidia, round. Antenna 1 0.3 × body length, flagellum 10–articulate, peduncle article 1 width less than 2 × article 2, accessory flagellum 1–articulate. Antenna 2 0.3 × body length, subequal in length with antenna 1, flagellum 8–articulate. Mandibular palp ratio of articles 1–3 1.0: 3.4: 2.0, article 2 with 11 long distal setae, article 3 with facial setae and 1 distal seta, incisors strongly dentate; left mandible with 10 raker spines, lacinia mobilis large, strongly toothed; right mandible with 11 raker spines, lacinia mobilis small, strongly dentate. Upper lip asymmetrically lobate, anterior margin setose. Lower lip inner lobes fused, bare; outer lobes with moderate gape, anterior margins setose. Maxilla 1 palp 2–articulate with 4 distal setae; outer plate with 9 distal robust setae. Maxilla 2 inner plate with 5 robust distal setae, 1 robust facial seta, and several slender facial setae; outer plate with 3 robust distal setae and 13 slender distal marginal setae. Maxilliped inner plates distal margin with a v-shaped indentation, with short robust setae; outer plate inner margin smooth, reaching 0.1 × palp article 1, with simple marginal setae, facial setae absent; palp article 4 subequal in length with article 3, distally acute.

Pereon. Coxae 1–4 relative widths 1.0: 1.2: 0.9: 1.6. Gnathopod 1 coxa smooth, bare, anterodistal margin produced, subquadrate, distal margin straight, posterior margin excavate, facial setae absent; basis distally expanded, anterior margin with 5 medium setae, posterior margin bare; ischium bare; carpus linear, distal length 11.2 × width, proximal margin smooth, distal margin with 2 short setae; propodus straight, palm dentate with 7 robust and 12 distal setae; dactylus smooth, reaching 0.4 × propodus length. Gnathopod 2 coxa broader than long, subequal in size with coxae 3, smooth, bare, anterior margin straight, anterodistally rounded, distal and posterior margins straight, facial setae absent; basis slightly posteriorly expanded, anterior margin with 7 long curved setae, distal margin with 2 long setae, posterior margin bare; ischium with 2 distal setae; carpus 0.3 × propodus length, curved, distally tapered, anterior margin smooth, with setulate-serrate marginal setae; propodus with 1 mediofacial setal row above midline, reaching 0.8 × propodus length, with 3 rows of submarginal setae, posterior margin smooth, palm convex with 2 large and several small tubercles; dactylus curved, proximal margin smooth with 2 setae, anterior margin distally acute, reaching 0.6 × propodus length. Pereopod 3 coxa length 1.3 × width, anterodistal corner overriding distal face of coxa 2 and extending below it, smooth, bare, anterior margin straight, distal margin oblique, posterior margin straight, facial setae absent. Pereopod 4 coxa smooth, bare, anterior margin produced, distal margin evenly rounded, posterior margin excavate, facial setae absent. Pereopods 5–7 coxae facial setae absent, bases width length ratios 1: 1.3, 1: 1.2, 1: 1.2, posterior margins bare; pereopods 5–6 bases posterior margins smooth, pereopod 7 basis posterior margin serrate.

Pleon. Epimera 1–2 with ventral setae, epimeron 3 bare; epimeron 3 posteroventral corner subquadrate. Uropods 1–2 relative lengths 1.0: 0.9. Uropod 1 peduncle 0.7 × inner ramus length, outer ramus 0.8 × inner ramus length; inner ramus with 4 robust setae on each margin; outer ramus with 4 robust setae. Uropod 2 peduncle 0.8 × inner ramus length, outer ramus 0.7 × inner ramus length; inner ramus with 4 robust setae; outer ramus with 3 robust setae. Uropod 3 missing. Telson 2.3 × longer than wide, with 2 simple facial setae, apex weakly tridentate.

**Female (sexually dimorphic characters).**

Gnathopod 1 carpus length 10.8 × width; basis anterior margin with 8 long setae. Gnathopod 2 basis distally expanded, anterior margin with 22 long curved setae, distal margin with 4 long curved setae; ischium with 3 long and 2 short distal setae; carpus distally truncate, anterior margin dentate; propodus palm with 1 row of submarginal setae.

**Figure 17.**
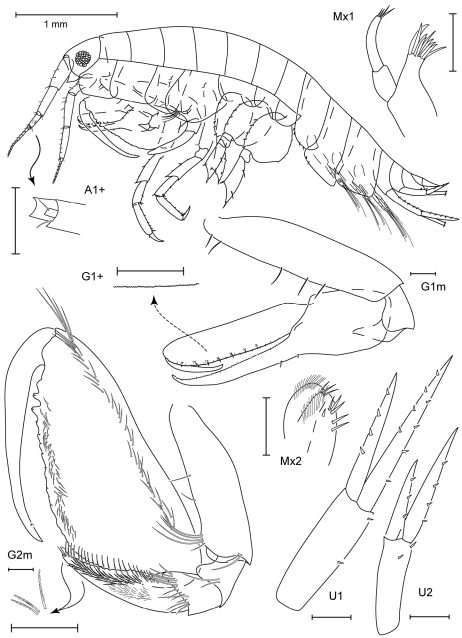
*Leucothoe togatta* sp. n., holotype male, 4.2 mm, RUMF-ZC-1778.

**Figure 18.**
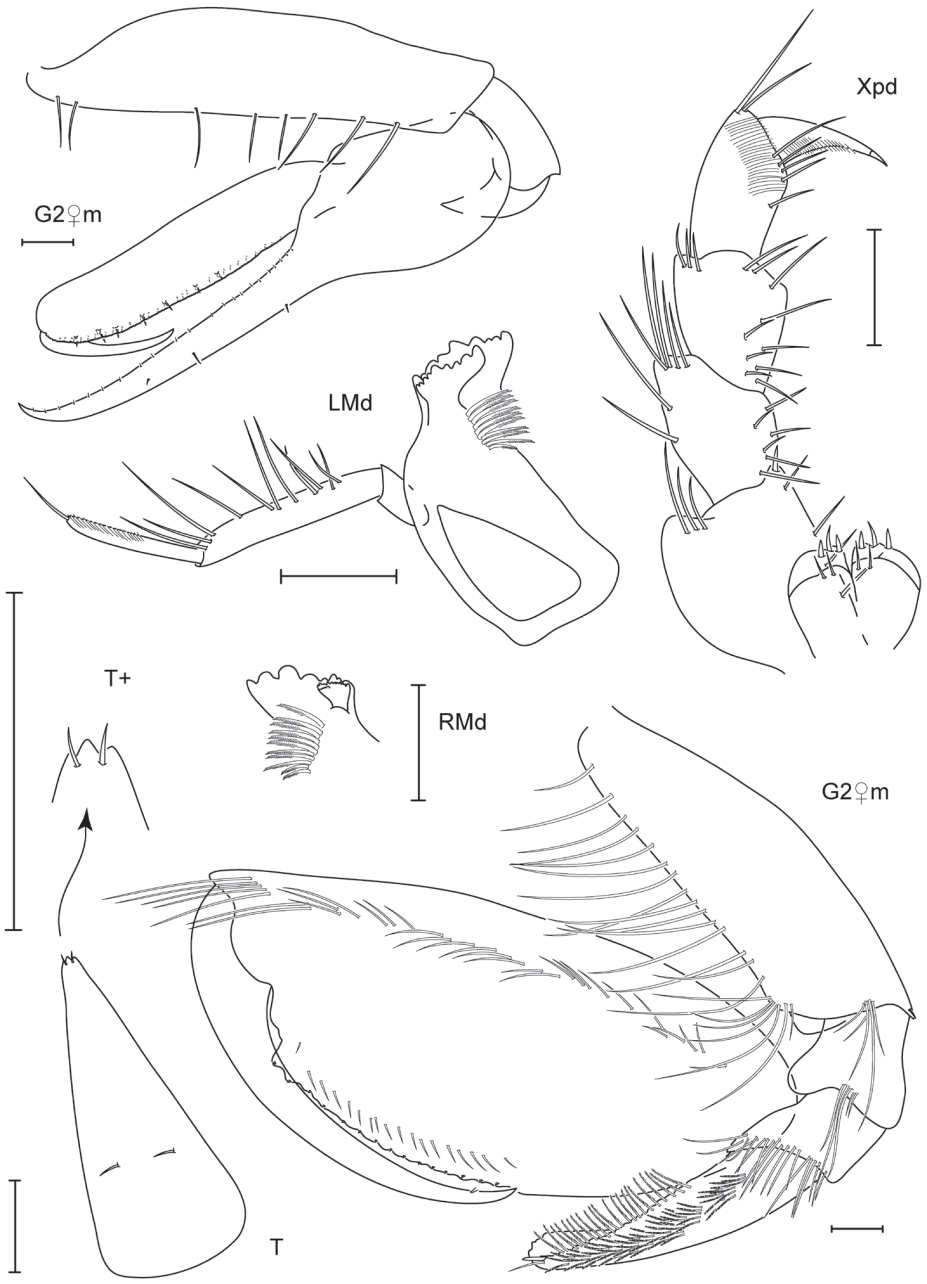
*Leucothoe togatta* sp. n., holotype male, 4.2 mm, RUMF-ZC-1778; paratype female, 5.4 mm, RUMF-ZC-1779.

##### Etymology.

After the Japanese word ‘togatta’, meaning ‘sharp’ and referring to the sharply pointed projection on the ventral cephalic keel. (Pronounced toe-ga-ta)

##### Ecology.

In canals of hard brown sponge, yellow inside with groups of small holes on top, ?*Jaspis* of Gray, 1867 (Figure 25C); and among coral rubble.

##### Relationships.

*Leucothoe togatta* sp. n. is similar to *Leucothoe ashleyae* Thomas & Klebba, 2006 and *Leucothoe saron* Thomas & Klebba, 2007 in having a rounded head margin, a 2–articulate maxilla 1 palp, high mediofacial setal row on gnathopod 2 propodus, wide pereopod 5–7 bases, and a tridentate telson. It also shares long curved setae on the distal margin of gnathopod 2 basis with *Leucothoe saron*. It differs from these species in having a ventral cephalic keel that is anteriorly excavate and anteroventrally quadrate with a projection. *Leucothoe togatta* sp. n. shares these keel characteristics with *Leucothoe amamiensis* White & Reimer, 2012a and *Leucothoe hashi* sp. n., but differs from *Leucothoe amamiensis* in the distally truncate gnathopod 2 carpus, from *Leucothoe hashi* in the robustness of gnathopod 1 propodus and length of gnathopod 1 dactylus, and from both species in the long curved setae on gnathopod 2 basis and robust row of submarginal setae on the gnathopod 2 propodus.

##### Remarks.

*Leucothoe togatta* sp. n. is white in color (Figure 23G). This species has been collected only on Yakushima Island and from both western and eastern coasts of Okinawa–jima Island, Okinawa.

##### Distribution.

East China Sea: Okinawa–jima Island (Okinawa), Yakushima Island (Kagoshima), Japan.

#### Leucothoe toribe sp. n.

urn:lsid:zoobank.org:act:430610B7-66FE-49E0-A683-C0CDD538E2D5

http://species-id.net/wiki/Leucothoe_toribe

Figures 19 20

##### Type material.

Holotype male, 3.3 mm RUMF-ZC-1784, Manza, Okinawa–jima Island, Okinawa, reef wall (26°30'15"N, 127°50'39"E), among coral rubble, 10–23 m, K.N. White and N.S. White, col., 8 February 2011 (KNWOkinawa29K). Paratype female, 3.8 mm RUMF-ZC-1785, same station data as holotype.

##### Type locality.

Manza, Okinawa–jima Island, Okinawa, Japan (26°30'15"N, 127°50'39"E).

##### Additional material examined.

4 specimens, RUMF-ZC-1786, KNWOkinawa22C; 5 specimens, NSMT-Cr 21902, KNWOkinawa23D; 2 specimens, RUMF-ZC-1787, KNWOkinawa23E; 1 specimen, NSMT-Cr 21903, KNWOkinawa25E; 4 specimens, RUMF-ZC-1788, KNWOkinawa27D; 6 specimens, NSMT-Cr 21904, KNWOkinawa29K; 6 specimens, RUMF-ZC-1789, KNWOkinawa30E; 1 specimen, RUMF-ZC-1790, KNWOkinawa33G; 12 specimens, NSMT-Cr 21905, KNWOkinawa39J.

##### Diagnosis (male).

Head anterodistal margin concave. Mandibular palp article 3 shorter than article 1; left mandible with 5 robust marginal setae. Maxilla 1 palp 1–articulate. Maxilliped inner plates with facial setae. Gnathopod 1 basis distally expanded, posterior margin with 5 short proximal setae; carpus robust; propodus curved, palm smooth with 22 short distal setae; dactylus reaching 0.2 × propodus length. Gnathopod 2 carpus distally truncate, spoon-like, with setulate-serrate setae; propodus with 3 mediofacial setal rows; dactylus elongate. Pereopods 5–6 coxae with facial setae; epimeron 3 posteroventral corner sinuous.

##### Description (male).

Head. Anterior margin transverse, anterodistal margin concave; ventral cephalic keel anterior margin transverse, anteroventral margin rounded, ventral margin excavate; eyes with more than 10 ommatidia, round. Antenna 1 0.4 × body length, flagellum 10–articulate, peduncle article 1 width less than 2 × article 2, accessory flagellum 1–articulate. Antenna 2 0.3 × body length, slightly shorter than antenna 1, flagellum 6–articulate. Mandibular palp ratio of articles 1–3 1.0: 2.6: 0.8, article 2 with 2 medium distal setae, article 3 with 1 distal seta, incisors weakly dentate; left mandible with 1 row of 5 and 1 row of 4 raker spines, with 5 robust marginal setae, lacinia mobilis large, strongly toothed; right mandible with 9 raker spines, lacinia mobilis small, weakly dentate. Upper lip asymmetrically lobate, anterior margin setose. Lower lip inner lobes fused, bare; outer lobes with moderate gape, anterior margins setose. Maxilla 1 palp 1–articulate with 4 distal setae; outer plate with 6 distal robust setae and 6 distal setae. Maxilla 2 inner plate with 3 robust distal and 3 robust marginal setae; outer plate with 4 robust distal setae and 4 sets of 2 slender distal marginal setae. Maxilliped inner plates distal margin with a v-shaped indentation, with short robust setae, facial setae present; outer plate inner margin smooth, reaching 0.3 × palp article 1, with simple marginal setae, facial setae absent; palp article 4 subequal in length with article 3, distally acute.

Pereon. Coxae 1–4 relative widths 1.0: 1.4: 1.1: 1.2. Gnathopod 1 coxa smooth, with tiny marginal setae, anterodistal margin produced, rounded, distal margin rounded, posterior margin excavate, facial setae absent; basis distally expanded, anterior margin with 1 short seta, anterodistal margin with several short slender setae, posterior margin with 5 setae; ischium bare; carpus linear, distal length 5.3 × width, proximal margin smooth, distal margin with 5 short setae; propodus curved, palm smooth with 22 distal setae; dactylus smooth, reaching 0.2 × propodus length. Gnathopod 2 coxa as long as broad, subequal in size with coxa 3, smooth, with tiny marginal setae, anterior margin straight, anterodistally subquadrate, distal and posterior margins straight, facial setae absent; basis distally expanded, anterior margin with 9 short setae, posterior margin with 1 short seta; ischium bare; carpus 0.2 × propodus length, curved, distally truncate, spoon-like, anterior margin smooth with setulate-serrate setae; propodus with 3 mediofacial setal rows, primary mediofacial setal row above midline, reaching 0.9 × propodus length, secondary mediofacial setal row with 9 setae, tertiary mediofacial setal row with 4 setae, posterior margin smooth, palmar corner pronounced, palm convex with 4 major tubercles; dactylus curved, proximal margin smooth with 2 setae, anterior margin distally subacute, reaching 0.7 × propodus length. Pereopod 3 coxa length 1.5 × width, anterodistal corner overriding distal face of coxa 2 and extending below it, smooth, with tiny marginal setae, anterior margin straight, distal margin slightly convex, posterior margin straight, facial setae absent. Pereopod 4 coxa smooth, with tiny marginal setae, anterior margin straight, distal margin produced, posterior margin excavate, facial setae absent. Pereopods 5–6 coxae facial setae present. Pereopod 7 coxa facial setae absent. Pereopods 5–7 bases width length ratios 1: 1.3, 1: 1.2, 1: 1.1, posterior margins smooth, setose.

Pleon. Epimera 1–2 with ventral setae, epimeron 3 bare; epimeron 3 posteroventral corner sinuous, rounded. Uropods 1–3 relative lengths 1.0: 0.7: 1.0. Uropod 1 peduncle subequal in length with inner ramus, outer ramus 0.8 × inner ramus length; inner ramus with 3 robust setae; outer ramus with 4 robust setae. Uropod 2 peduncle and outer ramus 0,7 × inner ramus length; inner and outer rami each with 3 robust setae. Uropod 3 peduncle and outer ramus subequal in length with inner ramus; inner ramus with 3 robust setae; outer ramus with 2 robust setae. Telson 2.5 × longer than wide, apex weakly tridentate.

**Female (sexually dimorphic characters).**

Gnathopod 2 basis linear, posterior margin bare; ischium with 5 posterodistal setae; carpus 0.4 × propodus length, distally tapered.

**Figure 19.**
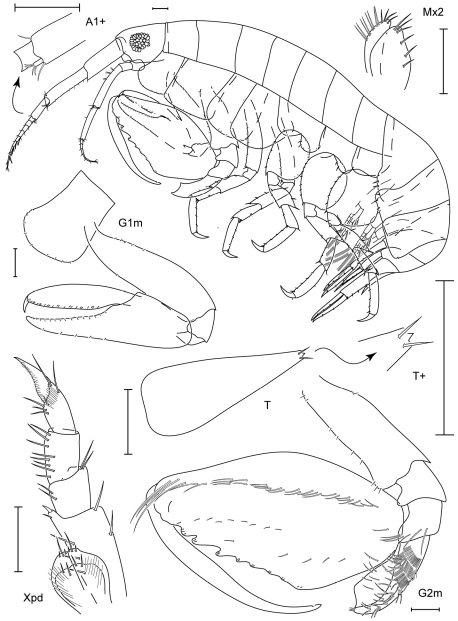
*Leucothoe toribe* sp. n., holotype male, 3.3 mm, RUMF-ZC-1784.

**Figure 20.**
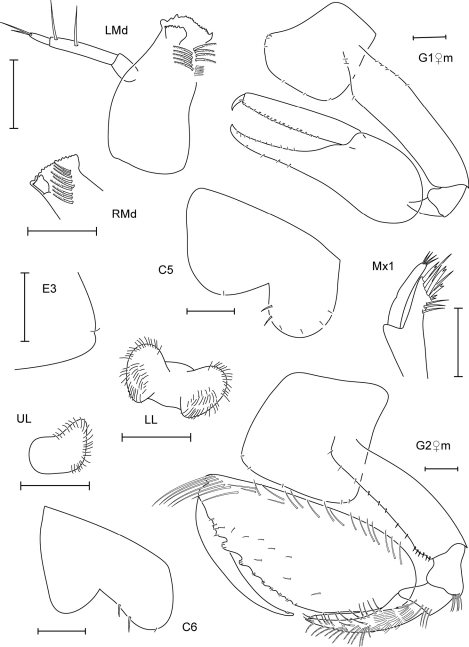
*Leucothoe toribe* sp. n., holotype male, 3.3 mm, RUMF-ZC-1784; paratype female, 3.8 mm, RUMF-ZC-1785.

##### Etymology.

After the Japanese word ‘toribe’, meaning ‘ladle’ and referring to the spoon-like carpus on gnathopod 2. (Pronounced toe-ree-bay)

##### Ecology.

In canals of green hard branching sponge, ?*Clathria (Thalysias) reinwardti* (Figure 25F); and among coral rubble.

##### Relationships.

*Leucothoe toribe* sp. n. is similar to *Leucothoe alata* (Barnard, 1959), *Leucothoe nagatai* Ishimaru, 1985, and *Leucothoe obuchii* White & Reimer, 2012a in having gnathopod 1 carpus reaching 0.1–0.2 × propodus length, and a curved gnathopod 1 propodus, but differs in having a 1–articulate maxilla 1 palp, broad pereopod 5–7 bases, and a sinuous posteroventral margin on epimeron 3. *Leucothoe toribe* sp. n. shares the spoon-like gnathopod 2 carpus with *Leucothoe alata* as well.

##### Remarks.

*Leucothoe toribe* sp. n. is faint pink in color, darkest along pereonite edges (Figure 23H). This species has been collected only on Yakushima Island, Kagoshima and from both western and eastern coasts of Okinawa–jima Island, Okinawa.

##### Distribution.

East China Sea: Okinawa–jima Island (Okinawa), Yakushima Island (Kagoshima), Japan.

#### Leucothoe zanpa sp. n.

urn:lsid:zoobank.org:act:74A3D66E-F5F5-4110-A799-A69E9BAE1028

http://species-id.net/wiki/Leucothoe_zanpa

Figures 21 22

##### Type material.

Holotype male, 3.2 mm, RUMF-ZC-1791, Zanpa Cape, Okinawa–jima Island, Okinawa, reef wall (26°26'27"N, 127°43'03"E), in canals of large white ball sponge, Tetillidae, 30 m, Daisuke Ueno, col., 26 February 2011 (KNWOkinawa34A). Paratype female, 3.8 mm, RUMF-ZC-1792, same station data as holotype.

##### Type locality.

Cape Zanpa, Okinawa–jima Island, Okinawa, Japan (26°26'27"N, 127°43'03"E).

##### Additional material examined.

12 specimens, RUMF-ZC-1793, KNWOkinawa34A; 13 specimens, NSMT-Cr 21906, KNWOkinawa34A; 3 specimens, RUMF-ZC-1794, KNWOkinawa34B; 2 specimens, NSMT-Cr 21907, KNWOkinawa34B.

##### Diagnosis (male).

Eyes rectangular. Antenna 1 0.6 × body length, peduncle article 1 width greater than 2 × article 2. Gnathopod 1 coxa about ½ as wide as coxa 2; basis proximally inflated, elongate; carpus and propodus elongate. Gnathopod 2 basis posterior margin with 2 long setae; ischium with 5 long posterodistal setae; propodus with 1 row of robust submarginal setae. Pereopod 5 coxa with facial setae; pereopods 5–7 bases very narrowly expanded. Uropod 3 peduncle 1.5 × inner ramus length. Telson apex truncate.

##### Description (male).

Head. Anterior margin excavate, anterodistal margin subquadrate; ventral cephalic keel anterior margin excavate, anteroventral margin rounded, ventral margin straight; eyes with more than 10 ommatidia, rectangular. Antenna 1 0.6 × body length, flagellum 6–articulate, peduncle article 1 width greater than 2 × article 2, accessory flagellum absent. Antenna 2 0.5 × body length, slightly shorter than antenna 1, flagellum 3–articulate. Mandibular palp ratio of articles 1–3 1.0: 2.0: 1.3, article 2 with 4–5 short distal setae, article 3 with 2 distal setae, incisors strongly dentate; left mandible with 8 raker spines, lacinia mobilis large, strongly toothed; right mandible with 12 raker spines, lacinia mobilis small, strongly dentate. Upper lip asymmetrically lobate, anterior margin setose. Lower lip inner lobes fused, bare; outer lobes with large gape, anterior margins setose. Maxilla 1 palp 2–articulate with 4 distal setae; outer plate with 4 distal robust setae and 7 distal setae. Maxilla 2 inner plate with 5 robust distal setae and 1 slender facial seta; outer plate with 4 robust distal setae, 7 distal and 5 proximal slender marginal setae. Maxilliped inner plates fused, distal margin with a v-shaped indentation, with short robust setae; outer plate inner margin tuberculate, reaching 0.1 × palp article 1, with simple marginal setae, facial setae present; palp article 4 subequal in length with article 3, distally acute.

Pereon. Coxae 1–4 relative widths 1.0: 1.9: 1.7: 1.7. Gnathopod 1 coxa smooth, with tiny marginal setae, anterior margin straight, anterodistal margin subquadrate, distal margin straight, posterior margin excavate, facial setae absent; basis proximally inflated, anterior margin with 4 setae, posterior margin with 2 setae; ischium bare; carpus linear, distal length 14.1 × width, proximal margin with denticles, distal margin bare; propodus curved, palm serrate with 13 distal setae; dactylus smooth, reaching 0.2 × propodus length. Gnathopod 2 coxa longer than broad, subequal in size with coxa 3, smooth, with tiny marginal setae, anterodistally rounded, distal margin straight, posterior margin straight, facial setae absent; basis linear, anterior margin with 5 short setae, posterior margin with 2 long setae; ischium with 5 long posterodistal setae; carpus 0.4 × propodus length, straight, distally tapered, anterior margin smooth; propodus with 1 mediofacial setal row displaced to midline, reaching 0.6 × propodus length, with 1 row of robust submarginal setae, posterior margin smooth, palm convex with many small tubercles; dactylus curved, proximal margin smooth, bare, anterior margin distally subacute, reaching 0.6 × propodus length. Pereopod 3 coxa length 0.8 × width, anterodistal corner overriding distal face of coxa 2, not extending below it, smooth, with tiny marginal setae, anterior margin evenly rounded, distal and posterior margins straight, facial setae absent. Pereopod 4 coxa smooth, with tiny marginal setae, anterior margin straight, distal margin evenly rounded, posterior margin straight, facial setae absent. Pereopod 5 coxa facial setae present, pereopods 6–7 coxae facial setae absent. Pereopods 5–7 bases width length ratios 1: 1.8, 1: 1.9, 1: 1.7, posterior margins smooth, setose.

Pleon. Epimera 1 and 3 bare, epimeron 2 with ventral setae; epimeron 3 posteroventral corner rounded. Uropods 1–3 relative lengths 1.0: 0.7: 0.8. Uropod 1 peduncle and outer ramus subequal in length with inner ramus; inner ramus with 1 robust seta; outer ramus lined with short marginal setae, with 5 robust setae. Uropod 2 peduncle 0.9 × inner ramus length, outer ramus 0.8 × inner ramus length; inner ramus with 2 robust setae; outer ramus lined with short marginal setae, with 2 robust setae. Uropod 3 peduncle 1.5 × inner ramus length, outer ramus subequal in length with inner ramus; inner ramus lined with short marginal setae, without robust setae; outer ramus with 3 robust setae. Telson 2.0 × longer than wide, apex truncate.

**Female (sexually dimorphic characters).**

Antenna 1 0.2 × body length. Antenna 2 0.3 × body length, subequal in length with antenna 1. Gnathopod 1 basis proximally wider than in male, posterior margin with 11 long setae. Gnathopod 2 basis anterior margin with 10 short setae.

**Figure 21.**
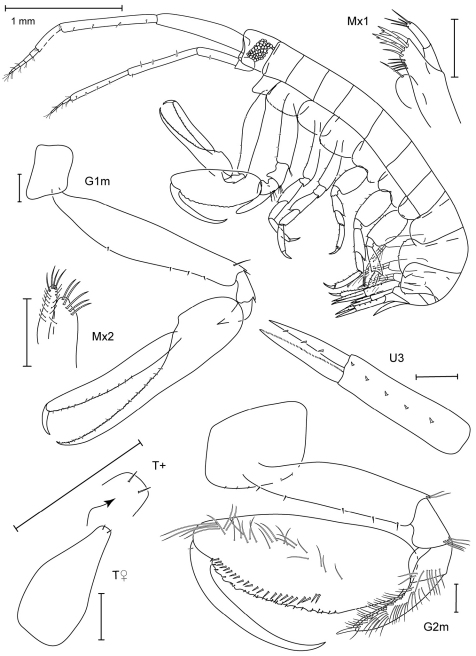
*Leucothoe zanpa* sp. n., holotype male, 3.2 mm, RUMF-ZC-1791.

**Figure 22.**
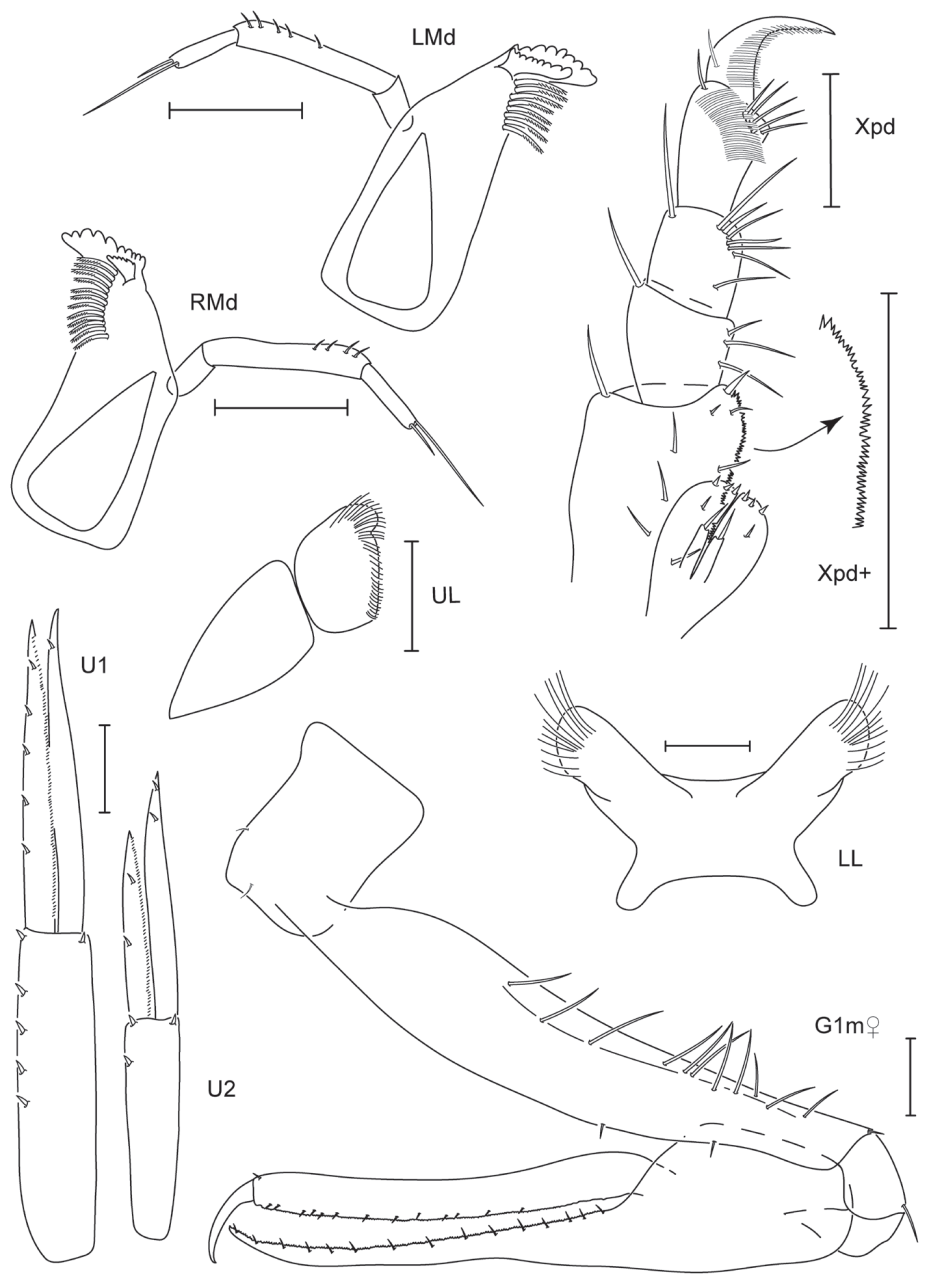
*Leucothoe zanpa* sp. n., holotype male, 3.2 mm, RUMF-ZC-1791; paratype female, 3.8 mm, RUMF-ZC-1792.

##### Etymology.

After the Japanese place name ‘Zanpa’, meaning ‘wave slicing’ and referring to the type locality.

##### Ecology.

In canals of large white ball sponge, Tetillidae (Figure 24I).

**Figure 23.**
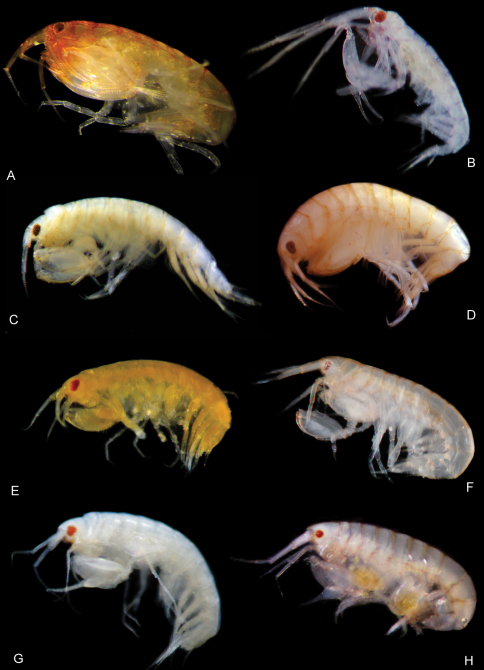
Color plate of new leucothoid amphipod species. **A** *Leucothoe akaoni* sp. n. **B** *Leucothoe zanpa* sp. n. **C** *Leucothoe lecroyae* sp. n. **D** *Leucothoe nurunuru* sp. n. **E** *Leucothoe ouraensis* sp. n. **F** *Leucothoe daisukei* sp. n. **G** *Leucothoe togatta* sp. n. **H** *Leucothoe toribe* sp. n.

**Figure 24.**
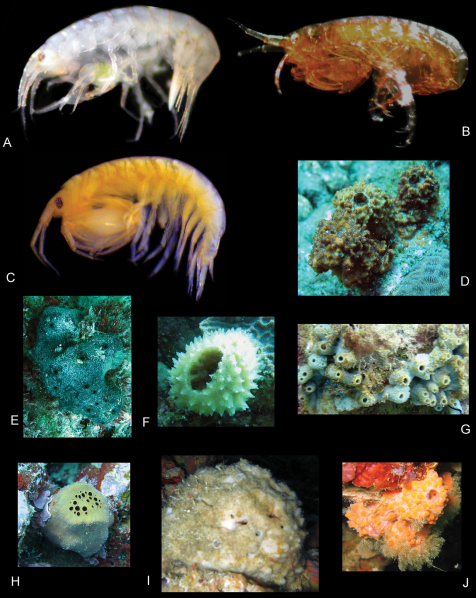
Color plate of new leucothoid amphipod species and sponge hosts. **A** *Leucothoe hashi* sp. n. **B** *Leucothoe bise* sp. n. **C** *Leucothoe nagatekubi* sp. n. **D** Axinellidae of Carter, 1875, RUMF-ZP-12 **E** ?*Axinyssa* of Lendenfeld 1897 **F** *Callyspongia* of Duchassaing and Michelotti 1864, RUMF-ZP-2 **G** *Haliclona* of Grant, 1836, RUMF-ZP-3 **H** *Rhabdastrella* of Thiele 1903, RUMF-ZP-1 I Tetillidae of Sollas 1886, RUMF-ZP-11 J Axinellidae of Carter, 1875.

**Figure 25.**
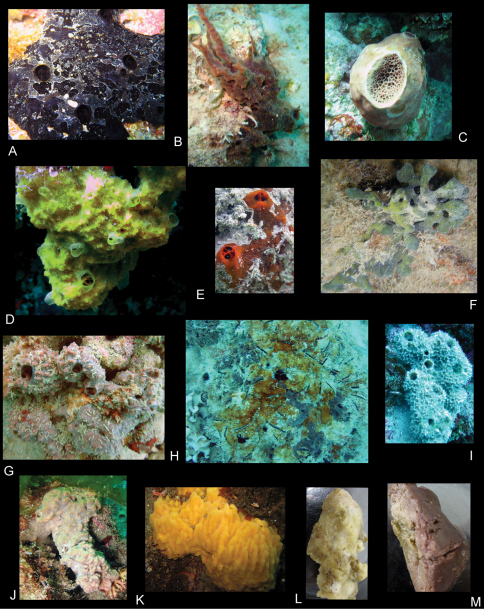
Color plate of sponge hosts. **A** Iotrochotidae of Dendy, 1922 (probably *Iotrochota* of Ridley 1884), RUMF-ZP-7 **B** *Mycale* of Gray, 1867, RUMF-ZP-8 **C** ?*Jaspis* of Gray, 1867 **D** orange encrusting sponge, *Clathria* of Schmidt, 1862, RUMF-ZP-5 **E** ?*Clathria (Thalysias) reinwardti* Vosmaer, 1880, RUMF-ZP-4 **F** *Tedania* of Gray, 1867 **G** *Mycale (Zygomycale) parishii* (Bowerbank, 1875), RUMF-ZP-9 **H** ?*Mycale* of Gray, 1867, RUMF-ZP-10 I Niphatidae of Van Soest, 1980 (probably *Niphates* of Duchassaing and Michelotti 1864), RUMF-ZP-6 **J** grey/purple hard sponge, *Clathria (Thalysias)* of Duchassaing and Michelotti 1864 **K** orange flame sponge, *Ciocalypta* of Bowerbank 1862 **L** orange stubby sponge, *Mycale* sp. **M** purple brown soft sponge, ?*Pericharax* of Poléjaeff 1883.

##### Relationships.

*Leucothoe zanpa* sp. n. is similar to *Leucothoe oboa* Karaman, 1971, *Leucothoe occulta* Krapp-Schickel, 1975, and *Leucothoe pachycera* Della Valle, 1893, in having antennae 1 peduncle article 1 width greater than 2 × the width of article 2, but differs in having an excavate anterior head margin, tuberculate maxilliped inner plate, setose gnathopod 2 basis posterior margin, pereopods 5–7 bases without facial setae, bare epimera 1 and 3, subquadrate epimeron 3 posteroventral margin, and truncate telson apex. *Leucothoe zanpa* sp. n. is similar to *Leucothoe elegans* White & Reimer, 2012a in the gracefully elongate gnathopod 1, but differs in eye shape, antennae length, and pereopod 5–7 bases widths.

##### Remarks.

*Leucothoe zanpa* sp. n. is white in color with faint purple stripes along pereonite edges (Figure 23B). This species is endemic to Zanpa Cape on the western coast of Okinawa–jima Island, Okinawa.

##### Distribution.

East China Sea: Okinawa–jima Island, Okinawa, Japan.

## Note

Sponge hosts for *Leucothoe elegans* White & Reimer, 2012a have been tentatively identified. These include: grey/purple hard sponge, *Clathria (Thalysias)* of Duchassaing and Michelotti 1864 (Figure 25J); dark red chimney sponge, Axinellidae (Figure 24D); orange flame sponge, *Ciocalypta* of Bowerbank 1862 (Figure 25K); purple brown soft sponge, ?*Pericharax* of Poléjaeff 1883 (Figure 25M); and orange stubby sponge, *Mycale* sp. (Figure 25L).

## Identification Key for sponge-dwelling Leucothoidae of the Ryukyu Archipelago (including Leucothoe elegans and Leucothoe vulgaris both White & Reimer 2012a)

**Table:** 

1	Antenna 1 length greater than 0.5 × body length (males), peduncle article 1 width greater than 2 × article 2; eyes rectangular; coxa 1 about ½ width of coxa 2, telson apex truncate	*Leucothoe zanpa*
–	Antenna 1 length less than 0.5 × body length, peduncle article 1 width less than 2 × article 2; eyes round or oval; coxa 1 subequal to or slightly smaller than coxa 2, telson apex tridentate, bidentate, or with strong point (rounded or sharp)	2
2	Gnathopod 1 propodus curved	3
–	Gnathopod 1 propodus straight	4
3	Gnathopod 1 palm without ornamentation; gnathopod 2 carpus distally truncate, spoon-like (male)	*Leucothoe toribe*
–	Gnathopod 1 palm dentate; gnathopod 2 carpus distally tapered	*Leucothoe elegans* White & Reimer, 2012a
4	Maxilliped outer plate tuberculate; gnathopod 1 dactylus 0.2 or less × propodus length	5
–	Maxilliped outer plate smooth; gnathopod 1 dactylus length greater than 0.2 × propodus length	8
5	Ventral cephalic keel anterior margin oblique, anteroventral margin with projection; gnathopod 1 basis distally expanded, propodus palm without large triangular teeth, dactylus reaching less than 0.1 × propodus length; telson apex bidentate	*Leucothoe ouraensis*
–	Ventral cephalic keel anterior margin transverse, anteroventral margin quadrate; gnathopod 1 basis linear, propodus palm with large triangular teeth, dactylus reaching 0.1–0.2 × propodus length; telson apex tridentate	6
6	Head anterior margin truncate, anterodistal margin quadrate with cusp; maxilliped outer plate with facial setae; gnathopod 1 basis posterior margin bare, distal margin setose; gnathopod 2 carpus distally tapered, propodus with 1 mediofacial setal row	*Leucothoe nagatekubi*
–	Head anterior margin round, anterodistal margin round; maxilliped outer plate without facial setae; gnathopod 1 basis posterior margin setose, distal margin bare; gnathopod 2 carpus distally truncate, propodus with 2 mediofacial setal rows	7
7	Antenna 1 accessory flagellum 1–articulate; mandibular palp article 3 shorter than article 1 and with 1 distal seta; maxilla 1 palp 1–articulate; gnathopod 1 basis anterior margin bare, carpus slender; pereopods 5–6 coxae without facial setae, pereopods 5–7 bases narrowly expanded	*Leucothoe hashi*
–	Antenna 1 accessory flagellum absent; mandibular palp article 3 subequal in length with article 1 and with 2 distal setae; maxilla 1 palp 2–articulate; gnathopod 1 basis anterior margin setose, carpus robust; pereopods 5–6 coxae with facial setae, pereopods 5–7 bases broadly expanded	*Leucothoe lecroyae*
8	Head anterodistal margin quadrate; gnathopod 2 propodus with 2 mediofacial setal rows; pereopods 5–7 bases narrowly expanded (length greater than 1.4 × width)	*Leucothoe bise*
–	Head anterodistal margin rounded or subquadrate; gnathopod 2 propodus with 1 mediofacial setal row; pereopods 5–7 bases broadly expanded (length 1.4 × width or less)	9
9	Ventral cephalic keel anteroventral margin with strong anterior projection; antenna 1 accessory flagellum 1–articulate; mandibular palp article 3 with 1 distal seta; gnathopod 2 propodus mediofacial setal row above midline; pereopod 7 basis posterior margin serrate	*Leucothoe togatta*
–	Ventral cephalic keel anteroventral margin subquadrate or quadrate with small projection; antenna 1 accessory flagellum absent; mandibular palp article 3 with 2 distal setae; gnathopod 2 propodus mediofacial setal row displaced to midline; pereopod 7 basis posterior margin smooth	10
10	Ventral cephalic keel anteroventral margin quadrate with small projection; maxilliped outer plate with facial setae; coxa 1 anterior margin serrate; gnathopod 1 ischium with posterior setae;	*Leucothoe akaoni*
–	Ventral cephalic keel anteroventral margin subquadrate; maxilliped outer plate without facial setae; coxa 1 anterior margin smooth; gnathopod 1 ischium bare	11
11	Mandibular palp article 2 with 15 distal setae; gnathopod 1 coxa anterior margin serrate, basis posterior margin bare; gnathopod 2 basis anterior margin with 10 setae, carpus reaching less than 0.4 × propodus length, propodus palm with large projections; pereopod 5 coxa without facial seta	*Leucothoe vulgaris* White & Reimer, 2012a
–	Mandibular palp article 2 with less than 10 distal setae; gnathopod 1 coxa anterior margin smooth, basis posterior margin setose; gnathopod 2 basis anterior margin with less than 10 setae, carpus reaching greater than 0.4 × propodus length, propodus palm with small projections; pereopod 5 coxa with facial setae	12
12	Antenna 1 flagellum 11–articulate; maxilla 1 palp 1–articulate, margins constricted; maxilliped inner plates with simple setae; gnathopod 1 carpus proximal margin smooth; gnathopod 2 basis posterior margin bare, carpus distally truncate; telson apex with strongly rounded point	*Leucothoe nurunuru*
–	Antenna 1 flagellum 6–articulate; maxilla 1 palp 1–articulate; maxilliped inner plates with simple and serrate setae; gnathopod 1 carpus proximal margin dentate; gnathopod 2 basis posterior margin setose, carpus distally tapered; telson apex tridentate	*Leucothoe daisukei*

## Discussion

Four of the *Leucothoe* species described here share the displaced gnathopod 2 propodus mediofacial row. This character is typically found in ascidian-dwelling species worldwide, suggesting that these species may also inhabit ascidian hosts, or that this character may not be an artifact of convergent evolution, as noted in White and Reimer (2012a). Six of the *Leucothoe* species described here have a small accessory flagellum on antenna 1. This character is unusual in leucothoid species and apparently much more common in Pacific species than in Caribbean species.

The currently recognized biogeographic boundaries of the Ryukyu Archipelago (Hikida and Ota 1997, Ota 1998) do not appear to apply to leucothoid amphipods in this region, although this will require further examination. Four species (*Leucothoe nagatekubi* sp. n., *Leucothoe nurunuru* sp. n., *Leucothoe ouraensis* sp. n., and *Leucothoe zanpa* sp. n.) were each found on only one island, while *Leucothoe hashi* sp. n. was collected throughout the entire archipelago. It is possible that these patterns in amphipod distributions are partly attributable to the ephemeral nature of their sponge hosts. Both sponge species and individuals in the Ryukyu Archipelago appear to be more prevalent in the winter months than in the summer.

Restricted distributional patterns were observed in *Leucothoe ouraensis* sp. n., which was collected only on the east coast of Okinawa–jima Island; *Leucothoe nagatekubi* sp. n., which was collected from one sponge host in caves at Mizugama on Okinawa–jima Island; *Leucothoe zanpa* sp. n., which was collected from only one sponge species at 30 meters at Zanpa Cape on Okinawa–jima Island; and *Leucothoe nurunuru* sp. n. was collected from only one sponge species at one location near Iriomote–jima Island. Interestingly, *Leucothoe akaoni* sp. n., *Leucothoe toribe* sp. n., and *Leucothoe togatta* sp. n. were all collected from Okinawa–jima Island in the mid-Ryukyus as well as from Yakushima Island, the northernmost island in the Ryukyus.

The host data collected here are invaluable in understanding the ecology of these amphipods. Sponges are often a preferred habitat for leucothoid amphipods, functioning as a food resource and as protection from predators (Thomas 1997, Thomas and Klebba 2007). Knowing the preferred habitat of amphipods will allow easier collection of additional specimens in the future. Furthermore, such data can also help us to understand the difference(s) between apomorphic and convergent morphological characters. Due to the difficulty in identifying sponges and lack of sponge specialists in the Ryukyu Archipelago, tentative identifications, photos, and descriptive data are provided here and pieces of host sponges have been deposited in the University of the Ryukyus Museum (Fujukan).

Most of the species reported here were collected from 1–4 sponge host species and from coral rubble. Presumably, these amphipods are inhabiting sponges in the crevices of the coral rubble. Perhaps the ephemeral nature of the sponges is also forcing the amphipods to adapt to new and available hosts, which may explain the higher number of species found in the genus *Leucothoe* than found in the anamixid clade of the Leucothoidae. *Leucothoe nagatekubi* sp. n., *Leucothoe nurunuru* sp. n., and *Leucothoe zanpa* sp. n. show higher specialization or host preference. Each of these species were collected from only one host at one location. With the exception of the large white ball sponge, Tetillidae (Figure 24I), individual sponges hosted only one leucothoid species at each location. *Leucothoe akaoni* sp. n., *Leucothoe daisukei* sp. n., *Leucothoe hashi* sp. n., and *Leucothoe zanpa* sp. n. were collected from the same white ball sponge at Zanpa Cape.

The very high diversity of new leucothoid species discovered in the Ryukyu Archipelago to date supports the theory of Roberts et al. (2002), stating that Indo–Pacific reefs are the most diverse areas in the world with high levels of endemicity.

## Supplementary Material

XML Treatment for Leucothoe
